# On-demand fluorescence control *via* self-assembly of amphiphilic acridone trimers†

**DOI:** 10.1039/d5ra04551g

**Published:** 2025-07-08

**Authors:** Isabelle Kolly, Simon M. Langenegger, Shi-Xia Liu, Robert Häner

**Affiliations:** a Department of Chemistry, Biochemistry and Pharmaceutical Sciences, W. Inäbnit Laboratory for Molecular Quantum Materials, WSS-Research Center for Molecular Quantum Systems, University of Bern Freiestrasse 3 3012 Bern Switzerland shi-xia.liu@unibe.ch robert.haener@unibe.ch

## Abstract

Self-assembly in aqueous media offers a promising strategy for the development of dynamic and adaptive materials. In this context, two new amphiphilic dicyanomethylenated acridone trimers have been synthesized and fully characterized. These molecules exhibit a reversible and tunable self-assembly behavior, forming spherical nanostructures in aqueous media. This self-assembly process is accompanied by distinct changes in their optical properties, remarkably an enhancement in fluorescence arising from restricted molecular torsion within the assembled structures. This study clearly demonstrates that crystalline-like properties can be achieved through solution-based self-assembly. Moreover, the length of the *N*-alkyl substituents on the acridone core plays a pivotal role in controlling both the self-assembly dynamics and the corresponding optical responses.

## Abstract 2

Mimicking crystalline-state properties is achieved by self-assembly of amphiphilic acridone trimers into nanostructures in aqueous media, showing enhanced fluorescence.

## Graphical abstract

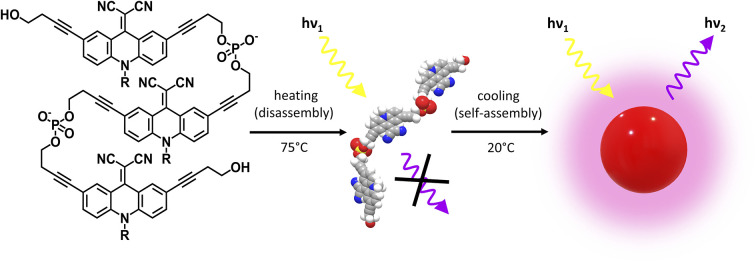

## Introduction

In materials science, the ability to achieve unique physical properties including rigidity, electrical conductivity, magnetic and optical behaviours, has been largely associated with the careful control of molecular motion in solid-state systems, where molecules are often tightly packed forming well-defined structures.^1–4^ In contrast, the inherent fluidity and dynamicity of the solution phase render it far more difficult to achieve the same level of control in molecular arrangement and torsion. However, our present work is challenging this paradigm by showcasing on-demand fluorescence control through supramolecular assembly in aqueous media. In the self-assembly process, non-covalent interactions such as hydrogen bonding, hydrophobic interactions, and van der Waals forces, steer the organization of molecules in a way that mimics the structural rigidity and specific functions typically associated with solid-state materials.^5–8^ With this approach, it is anticipated that dynamic, adaptive materials that exhibit solid-state-like properties considered unattainable in solution will be obtained.^9–12^

Our keen interest in acridone stems from its unique structure, which incorporates multiple functional groups for further derivatization. For instance, at the 10-position, the amine nitrogen is the nucleophile, capable of reacting with alkyl or aryl halides to form a C–N bond.^13,14^ Meanwhile, at the 9-position, the carbonyl group can undergo a Knoevenagel reaction with malononitrile, leading to the formation of a powerful electron-accepting dicyanomethylene group. Importantly, halogenation preferentially occurs at the reactive 2- and 7-positions, enabling subsequent C–C coupling reactions that are crucial to large extension of π-conjugation for enhancing electronic properties and stability of organic functional materials.^15–20^ As a result, a variety of acridone derivatives have been reported for diverse applications, including hole-transporting materials and host materials for organic light-emitting devices.^21–24^ Remarkably, once the carbonyl group is converted to the dicyanomethylene group, the resulting acridone derivatives exhibit crystallization-induced emission (CIE) behaviour in crystal structures. However, no fluorescence is observed in the amorphous powders. This behaviour originates from a flipping motion of the molecule, caused by the symmetrical twisting of the C=C(CN_2_) group from a planar structure. The potential energy barrier between the planar and twisted conformations is small and easily overcome at room temperature (*k*_BT_ ≈ 0.6 kcal mol^−1^),^25^ allowing the twisting motion to occur unless restricted. The restriction of this intramolecular torsional vibration through rigid crystal packing induces fluorescence and results in CIE behaviour.^25–29^ This raises the question: can ordered supramolecular nanostructures, formed *via* self-assembly in aqueous media, mimic the crystalline state and enhance emission?

**Fig. 1:**
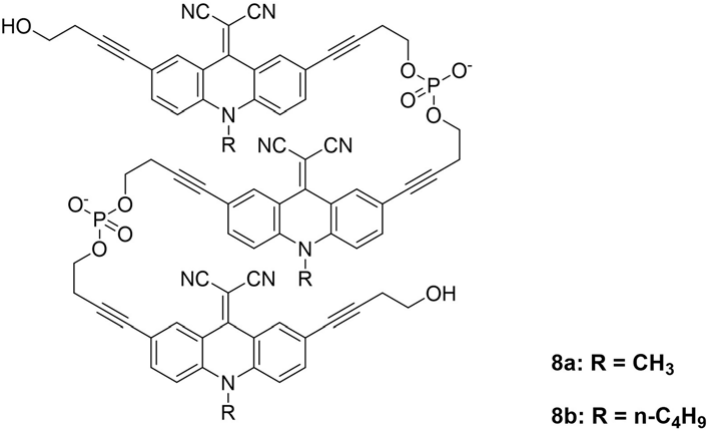
Chemical structures of two phosphodiester-linked acridone trimers.

Our group has previously demonstrated that phosphodiester-linked amphiphilic aromatic building blocks self-assemble in aqueous media to form supramolecular polymers (SPs).^30,31^ These SPs can adopt a range of nanostructures, including vesicles, fibers, sheets, ribbons, tubes, and toroids.^32–38^ Non-covalent interactions-driven self-assembly endows the resulting nanostructures with adaptable, reversible, and self-healable properties, rendering them highly promising for the construction of next-generation optoelectronics and biomaterials.^39–43^ Following this strategy, we present the synthesis and characterization of two new amphiphilic acridone trimers (Fig. 1), along with a comprehensive study of their self-assembly behaviour and photophysical properties using UV-vis absorption and fluorescence spectroscopy. The morphologies of the resulting supramolecular assemblies were visualized by atomic force microscopy (AFM). Our results also highlight the impact of *N*-substituent length on both the luminescence and the morphology of the nanostructures.

## Results and discussion

**Scheme 1:**
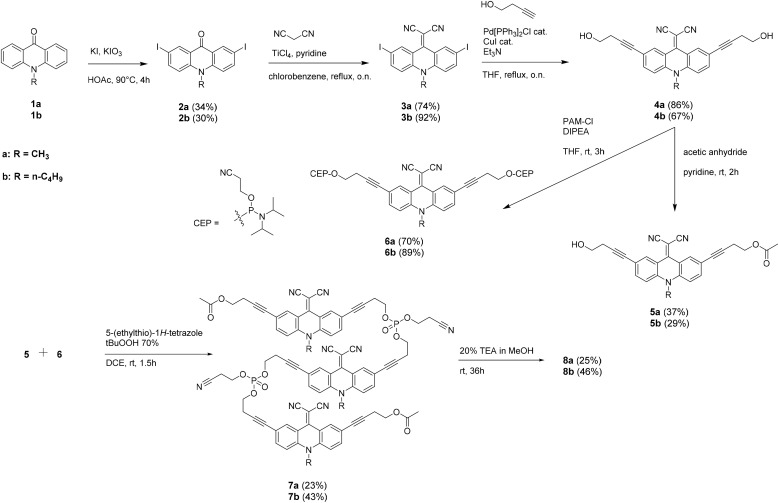
Synthesis of the trimers 8a/b.

The synthesis of the target compounds 8a/b is displayed in Scheme 1. Compounds 2a/b were obtained by iodination at 2,7-positions of acridone, following previously reported procedures.^26^ Subsequent Knoevenagel condensation with malononitrile yielded the dinitriles 3a/b in good yields. A Sonogashira cross-coupling reaction with 3-butyn-1-ol afforded the diols 4a/b. The monoprotected alcohols 5a/b were obtained by monoacetylation of 4a/b.

Phosphitylation of diol 4a/b resulted in the formation of bis-phosphoramidites 6a/b. The protected trimers 7a/b were synthesized by coupling bis-phosphoramidite 6a/b with the corresponding monoacetylated derivative 5a/b, using 5-(ethylthio)-1*H*-tetrazole as the activator and *tert*-butyl hydroperoxide as the oxidizer. Finally, phosphodiester-linked trimers 8a/b were obtained after deprotection of 7a/b in a triethylamine/methanol solution, followed by purification *via* reversed-phase HPLC and analysis by HRMS(ESI).

**Fig. 2:**
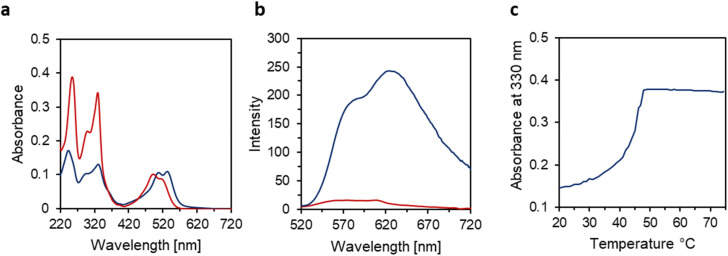
(a and b) UV-vis and fluorescence spectra before (red, 75 °C) and after slow cooling (blue, 20 °C) of 8a, *λ*_ex_ = 505 nm (c) absorbance at 330 nm during cooling from 75 °C to 20 °C of 8a (gradient 0.5 °C min^−1^). Conditions: aqueous solution with 3 μM trimer, 10 mM sodium phosphate buffer pH 7.2, 100 mM NaCl, 10 vol% ethanol.

Temperature-dependent UV-vis spectra of 8a and 8b were measured (Fig. 2a and ESI,† respectively) in an aqueous medium to study their self-assembly properties. At 75 °C, the disassembled trimer 8a exhibits prominent absorption maxima at 260 nm, 330 nm, 493 nm, and 520 nm (Fig. 2a, red curve), which closely resemble the spectra obtained in ethanol (ESI). The absorption bands below 400 nm are characteristic of 2,7-substituted acridone derivatives, while the weak, broad bands in the visible range are attributed to intramolecular charge transfer (ICT) transitions between the electron-donating amino group and the electron-accepting C=C(CN)_2_ groups.^27,44^ After cooling a solution of trimer 8a from 75 °C to 20 °C (cooling rate: 0.5 °C min^−1^), a hypochromic effect is observed in the UV region, accompanied by a bathochromic shifts of the ICT transitions by approximately 10–15 nm (Fig. 2a blue curve). This behaviour is attributed to thermally controlled self-assembly, leading to the formation of a supramolecular assembly.^45^ In comparison, trimer 8b shows a less pronounced hypochromic effect and bathochromic shift upon cooling (ESI), indicating that 8b self-assembles into a different ordered nanostructure than 8a.

To gain more insight into the self-assembly process, the absorbance at 330 nm was monitored during the cooling process. As shown in Fig. 2c, the absorbance curve for 8a follows a non-sigmoidal pattern, characteristic of a nucleation–elongation process, beginning at 49 °C. The reversible nature of the self-assembly process is further visualized *via* three consecutive heating/cooling cycles (ESI).

To further investigate the impact of the self-assembled nanostructures on the spectroscopic properties, temperature dependent fluorescence measurements were conducted (Fig. 2b). At 75 °C, 8a exhibits low fluorescence. Upon cooling to 20 °C, the assembled structures display a broad emission band with a maximum at 630 nm and a shoulder at 580 nm, along with a remarkable (*ca.* 15-fold) enhancement in fluorescence intensity compared to 75 °C. 8b shows similar behaviour, but with a slight blue shift in the emission band compared to 8a, resulting in a maximum at 560 nm and a shoulder at 506 nm (ESI). The observed blue shift aligns with previously reported emission properties of the corresponding monomer building blocks.^26^ This observation is very probably attributed to an “excimer-like” emission process occurring within in 8a-based supramolecular assemblies. The increase in fluorescence intensity for 8b-based nanostructures (*ca.* 4 fold), is much lower than that observed for 8a-based structures. However, the opposite behaviour was reported in their crystalline monomers.^26^ This suggests that torsional vibrations are more restricted in 8a-based supramolecular nanostructures due to more compact packing of the acridone cores compared to the 8b-based counterparts. Our findings demonstrate that on-demand fluorescence control in phosphodiester linked acridone trimers can be achieved through the self-assembly of building blocks with subtle structural variations.

Not surprisingly, 8a-based supramolecular assemblies exhibit a fluorescence quantum yield of 0.55%, which is approximately 10 times lower than the 5% quantum yield observed for the corresponding crystalline monomer.^26^ Nevertheless, the ordered supramolecular nanostructures of 8a in solution effectively suppress torsional vibrations, reinforcing the idea that supramolecular assembly in aqueous media provides a powerful strategy for achieving properties typically found only in highly ordered crystalline states.

**Fig. 3:**
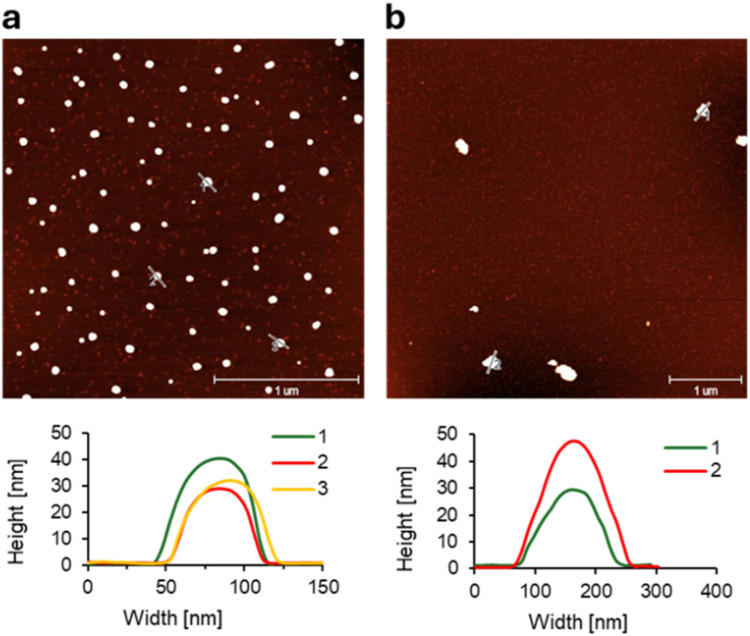
Microscopy measurements of oligomers (a) 8a and (b) 8b. AFM images with their corresponding cross sections. Conditions: see Fig. 2.

AFM was conducted to elucidate the morphology of the resulting supramolecular structures. The AFM images and height profiles reveal squeezed spherical nanostructures with a diameter of around 50 nm and a height of 30 nm for 8a (Fig. 3a). The squeezing of the spherical objects might be attributed to the AFM measurement technique.^46,47^ In contrast, the *n*-butyl-substituted trimer 8b forms larger, less uniform spherical structures, with an average diameter of 166 nm and a height of 20–70 nm (Fig. 3b). These findings are in agreement with the data obtained by DLS experiments (ESI).

## Conclusions

In summary, subtle modification in the substituent length of the acridone trimers results in significant spectroscopic changes of the resulting nanostructures formed through self-assembly in aqueous media. These changes manifest not only in the self-assembly/disassembly processes but also in the distinct changes of fluorescence intensity upon cooling. Supramolecular nanostructures containing 8a and 8b display enhanced fluorescence (15- and 4-fold, respectively), due to the restriction of molecular torsion within their nanostructures which resemble a crystalline state. By demonstrating that crystalline-state-like properties can be achieved through self-assembly in solution, this work opens a new frontier in material design.

## Conflicts of interest

There are no conflicts to declare.

## Supplementary Material

RA-015-D5RA04551G-s001

## Data Availability

The data supporting this article have been included as part of the ESI.†
