# Supplementary material for: On-demand fluorescence control via self-assembly of amphiphilic acridone trimers
Source: RSC Adv. 2025 Jul 8;15(29):23862–6. doi: 10.1039/d5ra04551g (PMC12236439; doi:10.1039/d5ra04551g)
Supplement: RA-015-D5RA04551G-s001 [file RA-015-D5RA04551G-s001.pdf]

Supplementary Information (SI) for RSC Advances.
This journal is © The Royal Society of Chemistry 2025

Electronic Supplementary Information

On-Demand Fluorescence Control via Self-Assembly of Acridone Trimers

Isabelle Kolly, Simon M. Langenegger, Robert Häner and Shi-Xia Liu*

Department of Chemistry, Biochemistry and Pharmaceutical Sciences, W. Inäbnit Laboratory for Molecular Quantum Materials and WSS-Research Center for Molecular Quantum Systems, University of Bern, Freiestrasse 3, 3012 Bern, Switzerland

Table of contents

1. General Methods 3
2. Synthesis of the Oligomers 3
    2.1. Organic Synthesis 3
    2.2. NMR Spectra 10
    2.3. HPLC traces 18
    2.4. MS Spectra 19
3. Preparation of the sample 24
4. Excitation Spectra 24
5. UV-vis & fluorescence spectra 25
6. Atomic force microscopy 27
    6.1. Zoom in 27
    6.2. Additional images 27
7. Dynamic light scattering 30
8. References 31

1. General Methods

All reagents and solvents were purchased from commercial sources and used without further purification. Compounds **1a** was purchased from commercial sources and **2b** was prepared according to previously published procedures.[1,2] Water was used from a Milli-Q system. NMR spectra were either obtained on a Bruker Avance III HD (300 MHz) or on a Bruker Avance II (400 MHz) spectrometer at 298 K Mass spectra were measured by the Analytical Research and Services (ARS) of the University of Bern, Switzerland, on a Thermo Fisher LTQ Orbitrap XL using Nano Electrospray Ionization (NSI). Dynamic light scattering (DLS) experiments were performed on a Malvern Zetasizer Nano Series instrument (λ = 633 nm) in particle size distribution (PSD) mode (number value). UV-Vis spectra were measured on a Jasco V-730 spectrophotometer using quartz cuvettes with an optical path of 1 cm. Fluorescence spectra were collected on a Jasco spectrophotometer FP-8300 using an excitation slit of 5 nm and emission slit of 5 nm. Supramolecular self-assembly was carried-out via thermal disassembly and reassembly. The sample solution was heated to 75 °C, then cooled with a gradient of 0.5 °C/min to 20 °C in a thermostat equipped with a Peltier. Atomic force microscopy (AFM) experiments were conducted under ambient conditions on a Nanosurf FlexAFM instrument using tapping mode. AFM samples were prepared on (3 aminopropyl)triethoxysilane (APTES)-modified mica sheets (Glimmer "V1", 20 mm x 20 mm, G250-7, Plano GmbH) according to published procedures.[3,4] The mica sheets were freshly cleaved and mounted with tape on top of a desiccator (3 L), before the desiccator was purged with argon. APTES (30 µL) was pipetted into an Eppendorf tube cap and Hünig's base (DIPEA) (10 µL) was added into a second cap. Both Eppendorf tube caps were placed at the bottom of the desiccator below the mica sheets, then the desiccator was closed. The mica sheets were left for one night in the desiccator to cure. Afterwards, the corresponding sample solution (30 µL) was pipetted onto the APTES-modified mica sheet. After an adsorption time of 10 min, the mica sheet was rinsed with Milli-Q water (1 mL), then dried under a stream of argon.

2. Synthesis of the Oligomers

2.1. Organic Synthesis

Preparation of compound **2a**

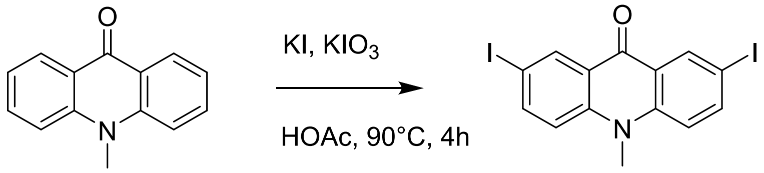

1a **2a**

2a (1.50 g, 7.17 mmol), KI (1.90 g, 11.47 mmol), and KIO_3 (1.23 g, 5.74 mmol) were heated at 90 °C for 4 h in acetic acid (40 mL). After the mixture was cooled to room temperature, the product was filtered off and successively washed by acetic acid and aqueous solution of sodium bisulfate. The crude product was further recrystallized by a mixture of ether/heptane to give the pure product as a yellow solid (1.10 g, 34%). ^1H NMR (300 MHz, CD_2Cl_2) δ 8.78 (d, *J* = 2.3 Hz, 2H), 8.00 (dd, *J* = 8.9, 2.3 Hz, 2H), 7.35 (d, *J* = 9.0 Hz, 2H), 3.84 (s, 3H). HRMS-NSI (*m/z*): $[M+H]^+$ calcd for $C_{14}H_{10}ONI_2$, 461.8846; found, 461.8845.

Preparation of compound **3a**

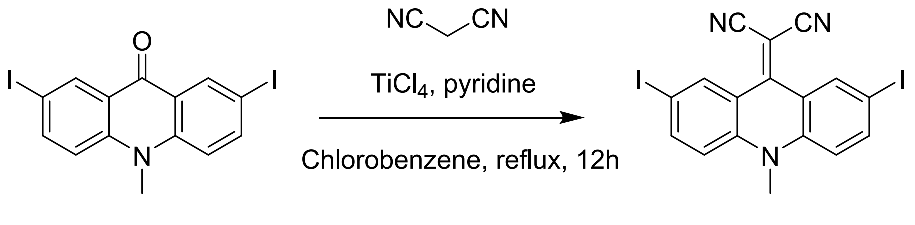

2a (1.5 g, 3.25 mmol) was dissolved in chlorobenzene (150 mL), then malononitrile (6.45 g, 97.61 mmol), pyridine (13.13 mL, 162.68 mmol) and $TiCl_4$ (3.57 mL, 32.54 mmol) were added. The mixture was stirred for 15 min at room temperature, then heated at reflux for 12 h. The reaction was performed under argon atmosphere. After cooling to room temperature, the reaction mixture was filtered over celite, the filtrate was concentrated and purified by column chromatography on silica gel (CH_2Cl_2/toluene 9:1) to give the product as a red solid (1.22 g, 74%). ^{1}H NMR (300 MHz, DMSO-d_6) δ 8.51 – 8.36 (m, 2H), 7.82 (dd, *J* = 1.8, 0.9 Hz, 2H), 7.41 (ddd, *J* = 8.2, 6.2, 2.0 Hz, 2H), 3.93 (s, 3H). HRMS-NSI (*m/z*): $[M+H]^+$ calcd for $C_{17}H_9I_2N_3$, 508.8886; found, 509.8950.

Preparation of compound **4a**

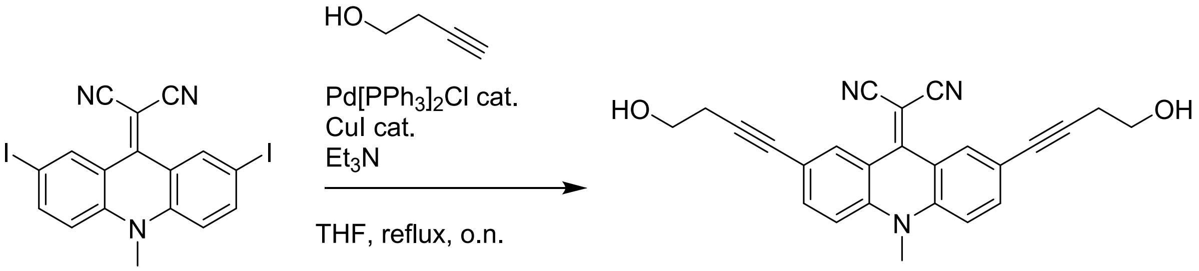

3a (200 mg, 0.39 mmol), $Pd[PPh_3]_2Cl_2$ (8 mg, 0.01 mmol, 0.03 eq.) and CuI (3 mg, 0.02 mmol, 0.04 eq.) were added to a round bottom flask, evacuated and refilled with argon three times. Anhydrous THF (30 mL) and degassed triethylamine (TEA) (4 mL) were added, followed by the addition of but-3-yn-1-ol (0.12 mL, 1.57 mmol) and the reaction mixture was heated at reflux overnight. After completion, the mixture was cooled to room temperature, diluted with CH_2Cl_2 (100 mL) and filtered through celite. The filtrate was washed with 10% citric acid solution (40 mL) and once with a saturated $NaHCO_3$ solution (50 mL), dried over $NaSO_4$, filtrated and dried in vacuo. The residue was then purified by column chromatography on silica gel (gradient v:v, CH_2Cl_2/toluene/ isopropanol 89:10:1 → 85:10:5) to give the pure product as a red solid (133 mg, 86%). ^{1}H NMR (300 MHz, CD_2Cl_2) δ 8.47 (d, *J* = 1.8 Hz, 2H), 7.73 (dd, *J* = 8.9, 1.9 Hz, 2H), 7.41 (d, *J* = 8.9 Hz, 2H), 4.01 – 3.65 (m, 7H), 2.71 (t, *J* = 6.3 Hz, 4H), 1.84 (t, *J* = 6.3 Hz, 2H). HRMS-NSI (*m/z*): $[M+H]^+$ calcd for $C_{25}H_{19}N_3O_2$, 349.1500; found, 394.1544.

Preparation of compound **5a**

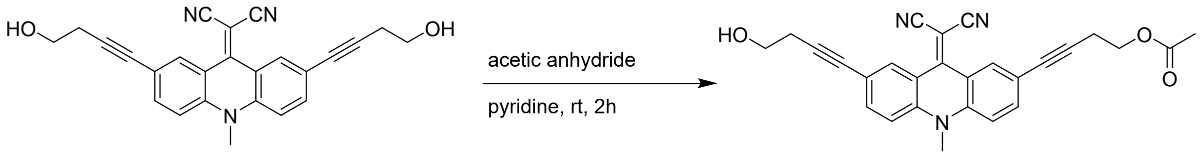

4a **5a**

4a (180 mg, 0.457 mmol) was dissolved in pyridine (6 mL) under argon. A 2 M solution of acetic anhydride in pyridine (0.23 mL, 0.457 mmol) was added dropwise over 10 min to the dissolved compounds while cooling the reaction mixture. The reaction was then stirred at rt for 2 h. Then the mixture was diluted in CH_2Cl_2 (30 mL) and washed once with 0.5M HCl (30 mL), saturated $NaHCO_3$ (40 mL) and brine (30 mL). The organic phase was dried over $MgSO_4$ and dried in vacuo. The crude product was purified by column chromatography on silica gel (v:v, CH_2Cl_2/toluene/ isopropanol 88:10:2) to give the pure product (74 mg, 37%). ^{1}H NMR (300 MHz, CD_2Cl_2) δ 8.47 (t, *J* = 2.1 Hz, 2H), 7.72 (ddd, *J* = 8.9, 3.6, 1.9 Hz, 2H), 7.41 (d, *J* = 8.9 Hz, 2H), 4.25 (t, *J* = 6.8 Hz, 2H), 3.91 – 3.74 (m, 5H), 2.79 (t, *J* = 6.8 Hz, 2H), 2.71 (t, *J* = 6.3 Hz, 2H), 2.08 (s, 3H), 1.83 (t, *J* = 6.3 Hz, 1H). HRMS-NSI (*m/z*): $[M+H]^+$ calcd for $C_{27}H_{21}N_3O_3$, 436.1656; found, 436.1658.

Preparation of compound **6a**

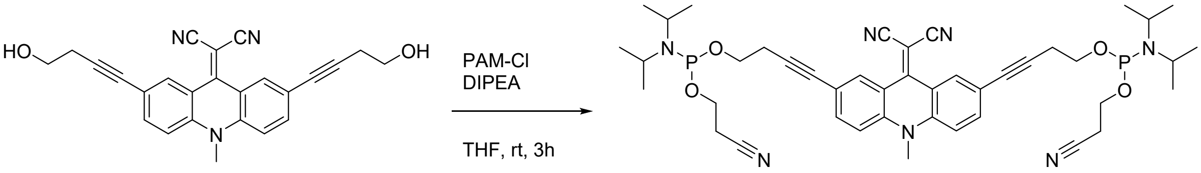

4a **6a**

4a (100 mg, 0.254 mmol) was dissolved in dry THF (10 mL) and DIPEA (0.5 mL). 2-Cyanoethyl N,N-diisopropylchlorophosphoramidite (150 mg, 0.635 mmol) was added dropwise at room temperature and the reaction was stirred for 3 h under argon. The reaction mixture was concentrated under reduced pressure and the resultant crude product was purified by a short column on silica gel (v:v EtOAc/heptane 2:1) to give the pure product (141 mg, 70%). 1% Et_3N was always added for the column and TLC eluent to prevent degradation of the compound. The product should be stored under argon in the freezer. ^{1}H NMR (300 MHz, $CDCl_3$) δ 8.47 (d, *J* = 1.8 Hz, 2H), 7.68 (dd, *J* = 8.9, 1.9 Hz, 2H), 7.35 (d, *J* = 8.9 Hz, 2H), 3.97 – 3.71 (m, 11H), 3.62 (dq, *J* = 10.2, 6.8 Hz, 4H), 2.75 (t, *J* = 6.8 Hz, 4H), 2.66 (td, *J* = 6.1, 1.4 Hz, 4H), 1.20 (d, *J* = 6.8 Hz, 24H). ^{31}P NMR (121 MHz, $CDCl_3$) δ 148.10.

Preparation of compound **7a**

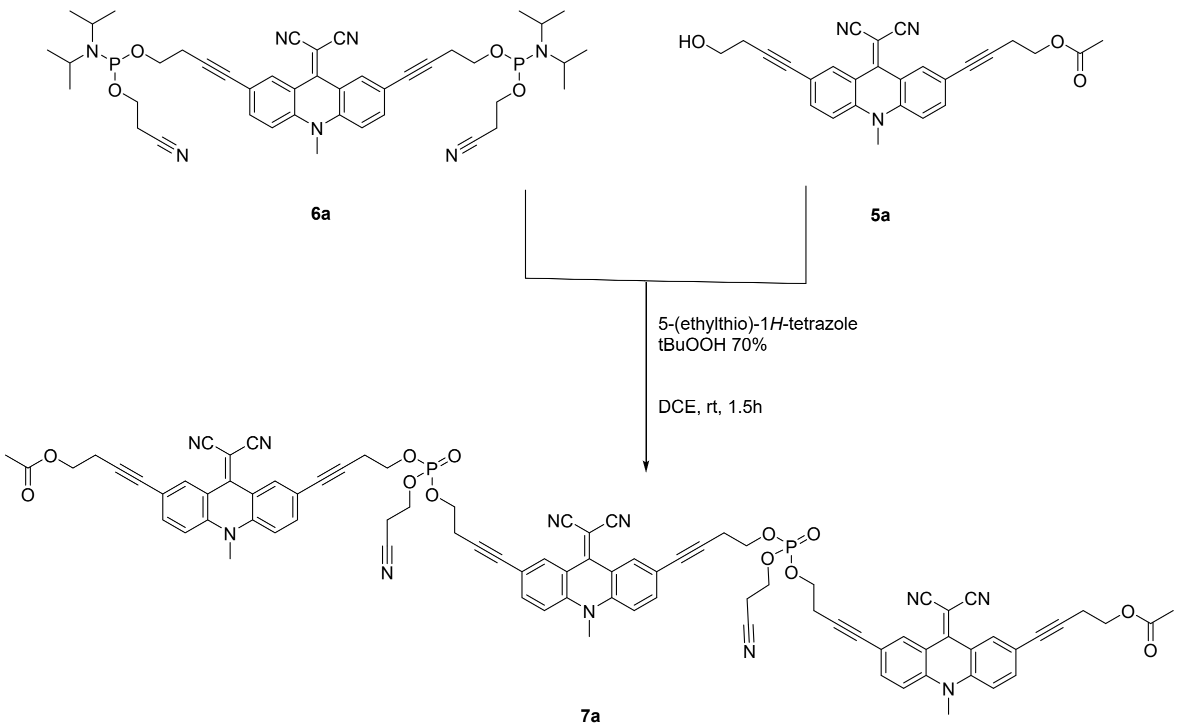

A solution of ethyl-thio-tetrazole (20 mg, 0.151 mmol, 4 eq.) in 1,2-dichloroethane (0.3 mL) was added under argon atmosphere to a solution of **6a** (30 mg, 0.038 mmol) in DCE (0.3 mL). **5a** (33 mg, 0.076 mmol, 2 eq.) was dissolved in DCE (0.6 mL) and added to the activated compound **6a**. The reaction was then stirred at rt for 1.5 h. Then tBuOOH solution (70% in water, 31 µL, 0.23 mmol) was added and the reaction mixture was further stirred for 20 min. Then the reaction mixture was diluted with 20 mL $CHCl_3$, washed once with saturated $NaHCO_3$ (20 mL) and brine (20 mL). The organic layer was dried with $MgSO_4$ and concentrated. The residue was further purified by preparative TLC on silica (v:v CH_2Cl_2/toluene/ isopropanol 85:10:5) to give the desired product (13 mg, 23%). ^1H NMR (300 MHz, $CDCl_3$) δ 8.38 – 8.13 (m, 6H), 7.71 – 7.54 (m, 6H), 7.32 (d, *J* = 8.9 Hz, 3H), 7.25 – 7.15 (m, 3H), 4.45 – 4.23 (m, 16H), 3.74 (d, *J* = 16.1 Hz, 9H), 2.90 (t, *J* = 6.2 Hz, 8H), 2.83 – 2.75 (m, 8H), 2.12 (s, 6H). ^{31}P NMR (121 MHz, $CDCl_3$) δ -2.32. HRMS-NSI (*m/z*): $[M+Na]^+$ calcd for $C_{85}H_{65}O_{12}N_{11}P_2$, 1516.4182; found, 1516.4218.

Preparation of compound **8a**

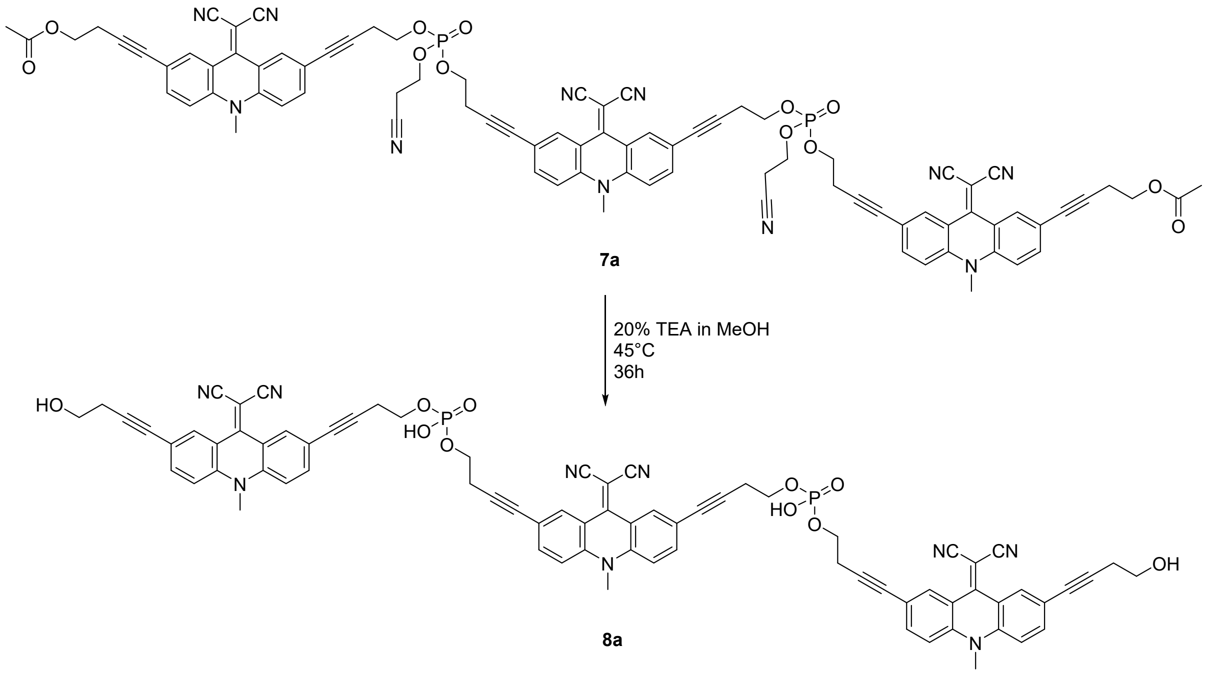

7a (5 mg) was dissolved in a solution of 20% triethylamine (TEA) in methanol (5 mL) and stirred at 45°C for 36 h. The sample was further lyophilized and purified by RP-HPLC (Shimadzu LC-20AT, ReproSil 100 C8, 5,0 μm, 250×4 mm) at 40 °C with a flow rate of 1 mL/min. Solvent A: aqueous 2.1 mM TEA / 25 mM 1,1,1,3,3,3-hexafluoropropan-2-ol (HFIP) pH 8; solvent B: acetonitrile; gradient: B [%] t_R [min]: 10 (0), 10 (2), 60 (20). The purified trimer was dissolved in EtOH and the absorbance of the compound was measured to determine the concentration of the stock solution. The Beer-Lambert law was applied to determine the concentrations. The molar absorption coefficient was determined to be: ε(8a, 504 nm): 44'300 $L\cdot mol^{-1}\cdot cm^{-1}$. The final product is a red solid (1 mg, 25%). HRMS-NSI (*m/z*): $[M-2H]^{2-}$ calcd for $C_{75}H_{53}N_9O_{10}P_2$, 650.6701; found, 650.6679.

Preparation of compound **3b**

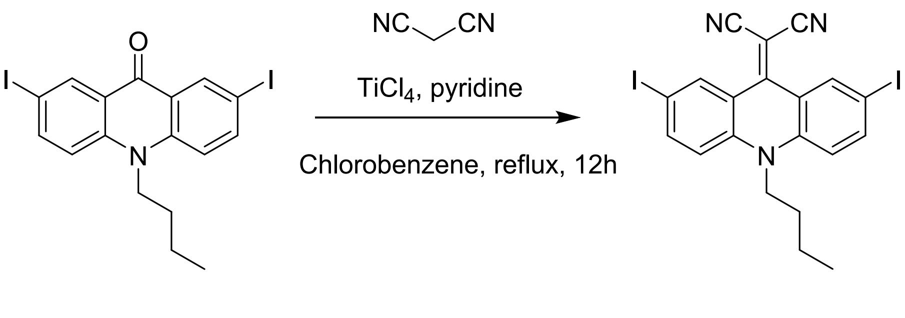

2b **3b**

2b (3 g, 5.96 mmol) was dissolved in chlorobenzene (300 mL), then malononitrile (11.82 g, 178.88 mmol), pyridine (24 mL, 298.14 mmol) and $TiCl_4$ (6.54 mL, 59.63 mmol) were added. The mixture was stirred for 15 min at room temperature, then heated reflux for 12 h. The reaction was performed under argon atmosphere. After cooling to room temperature, the reaction mixture was filtered over celite and washed with chloroform. The filtrate was concentrated and purified by column chromatography on silica gel (CH_2Cl_2/heptane gradient from 1:2 → 1:0) to give the product as a red

solid (3.04 g, 92%). ^{1}H NMR (300 MHz, $CDCl_3$) δ 8.76 (d, *J* = 2.0 Hz, 2H), 7.93 (dd, *J* = 9.1, 2.0 Hz, 2H), 7.19 (d, *J* = 9.1 Hz, 2H), 4.32 – 4.13 (m, 2H), 1.88 (p, *J* = 7.6 Hz, 2H), 1.51 (d, *J* = 7.4 Hz, 2H), 1.07 (t, *J* = 7.3 Hz, 3H). HRMS-NSI (*m/z*): $[M+H]^+$ calcd for $C_{20}H_{15}I_2N_3$, 551.9428; found, 551.9423.

Preparation of compound **4b**

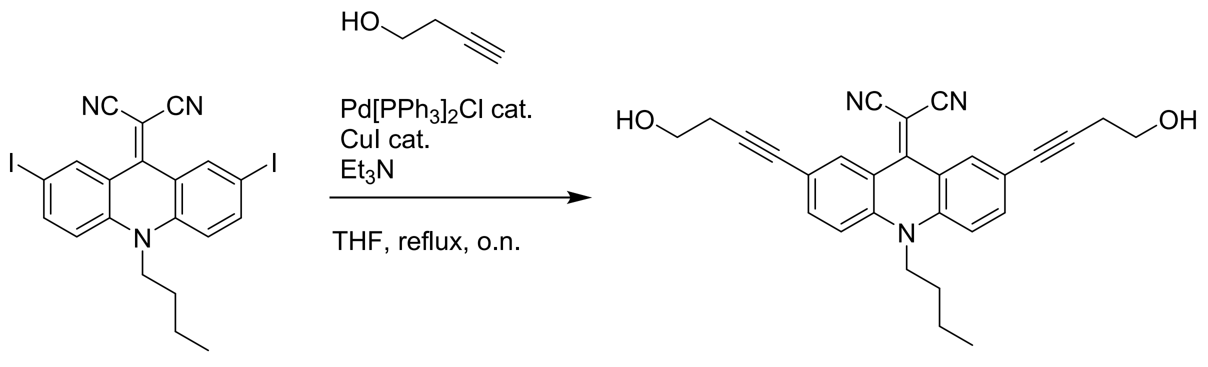

3b **4b**

3b (2.7 g, 4.9 mmol), $Pd[PPh_3]_2Cl_2$ (103 mg, 0.15 mmol, 0.03 eq.) and CuI (37 mg, 0.20 mmol, 0.04 eq.) were added to a round bottom flask, evacuated and refilled with argon three times. Anhydrous THF (200 mL) and degassed TEA (50 mL) were added, followed by the addition of but-3-yn-1-ol (1.48 mL, 1.37 mmol) and the reaction mixture was heated at reflux overnight. After completion, the mixture was cooled to room temperature, diluted with CH_2Cl_2 (200 mL) and filtered through celite. The filtrate was washed with 10% citric acid solution (200 mL) and once with a saturated $NaHCO_3$ solution (200 mL), dried over $NaSO_4$, filtrated and dried in vacuo. The residue was then purified by column chromatography on silica gel (gradient v:v, CH_2Cl_2/toluene/ isopropanol 89:10:1 → 80:10:10) to give the pure product as a red solid (1.42 mg, 67%). ^{1}H NMR (300 MHz, $CDCl_3$) δ 8.54 (d, *J* = 1.9 Hz, 2H), 7.69 (dd, *J* = 8.9, 1.9 Hz, 2H), 7.37 (d, *J* = 9.0 Hz, 2H), 4.32 – 4.20 (m, 2H), 3.84 (q, *J* = 6.3 Hz, 4H), 2.72 (t, *J* = 6.3 Hz, 4H), 1.91 (t, *J* = 7.8 Hz, 2H), 1.82 (t, *J* = 6.3 Hz, 2H), 1.51 (s, 2H), 1.08 (t, *J* = 7.3 Hz, 3H). HRMS-NSI (*m/z*): $[M+H]^+$ calcd for $C_{28}H_{26}O_2N_3$, 436.2020; found, 436.2024.

Preparation of compound **5b**

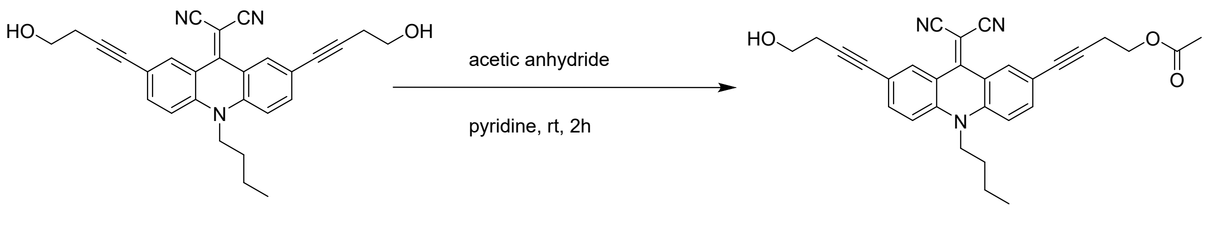

4b **5b**

4b (500 mg, 1.15 mmol) was dissolved in pyridine (16 mL) under argon. A 2 M solution of acetic anhydride in pyridine (0.57 mL, 0.117 mmol) was added dropwise over 10 min to the dissolved compounds while cooling the reaction mixture. The reaction was then stirred at rt for 2 h. Then the mixture was diluted in CH_2Cl_2 (50 mL) and washed once with 0.5M HCl (50 mL), saturated $NaHCO_3$ (50 mL) and brine (50 mL). The organic phase was dried over $MgSO_4$ and dried in vacuo. The crude product was purified by column chromatography on silica gel (v:v, CH_2Cl_2/toluene/ isopropanol 89:10:1) to give the pure product (160 mg, 29%). ^{1}H NMR (300 MHz, $CDCl_3$) δ 8.53 (dd, *J* = 3.2, 1.8 Hz, 2H), 7.68 (ddd, *J* = 9.0, 3.6, 1.9 Hz, 2H), 7.37 (d, *J* = 9.0 Hz, 2H), 4.37 – 4.16 (m, 4H), 3.84 (q, *J* = 6.3 Hz, 2H), 2.75 (dt, *J* =

16.1, 6.6 Hz, 4H), 2.11 (s, 3H), 1.96 – 1.83 (m, 2H), 1.79 (t, *J* = 6.3 Hz, 1H), 1.50 (d, *J* = 7.2 Hz, 2H), 1.08 (t, *J* = 7.3 Hz, 3H). HRMS-NSI (*m/z*): $[M+H]^+$ calcd for $C_{30}H_{28}O_3N_3$, 478.2125; found, 478.2122.

Preparation of compound **6b**

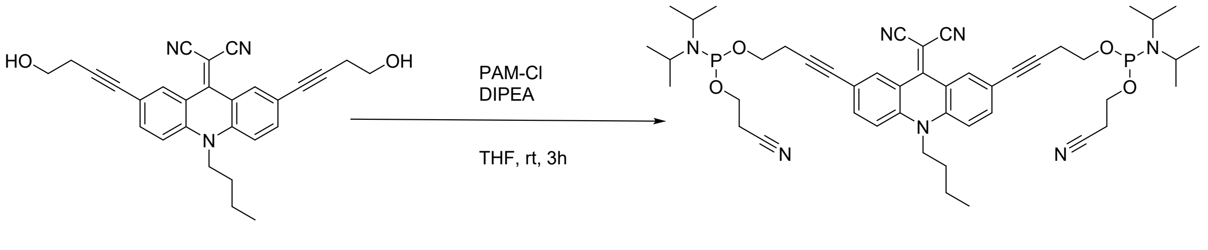

4b **6b**

4b (100 mg, 0.23 mmol) was dissolved in dry THF (10 mL) and DIPEA (0.5 mL).). 2-Cyanoethyl N,N-diisopropylchlorophosphoramidite (163 mg, 0.689 mmol) was added dropwise at room temperature and the reaction was stirred for 3 h under argon. The reaction mixture was concentrated under reduced pressure and the resultant crude product was purified by a short column on silica gel (v:v EtOAc/heptane 2:1) to give the pure product (171 mg, 89%). 1% Et_3N was always added for the column and TLC eluent to prevent degradation of the compound. The product should be stored under argon in the freezer. ^1H NMR (300 MHz, $CDCl_3$) δ 8.50 (d, *J* = 1.8 Hz, 2H), 7.67 (dd, *J* = 9.0, 1.9 Hz, 2H), 7.36 (d, *J* = 9.0 Hz, 2H), 4.31 – 4.19 (m, 2H), 4.01 – 3.72 (m, 8H), 3.66 – 3.49 (m, 4H), 2.83 – 2.57 (m, 8H), 1.90 (s, 2H), 1.51 (s, 2H), 1.20 (dd, *J* = 6.8, 1.0 Hz, 24H), 1.07 (t, *J* = 7.4 Hz, 3H). ^{31}P NMR (121 MHz, $CDCl_3$) δ 148.09.

Preparation of compound **7b**

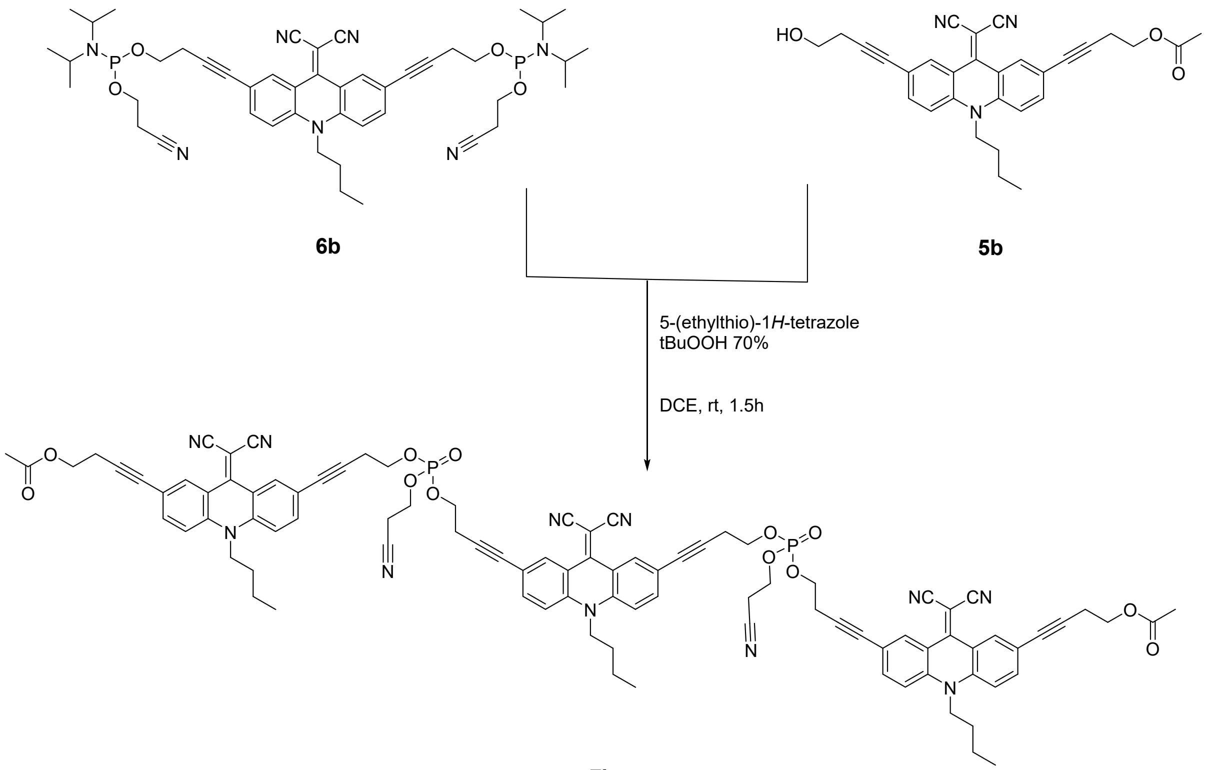

7b

A solution of ethyl-thio-tetrazole (19 mg, 0.144 mmol, 4 eq.) in DCE (0.3 mL) was added under argon atmosphere to a solution of **6b** (30 mg, 0.036 mmol) in DCE (0.3 mL). **5b** (34 mg, 0.072 mmol, 2 eq.)

was dissolved in DCE (0.6 mL) and added to the activated compound **6b**. The reaction was then stirred at rt for 1.5 h. Then tBuOOH solution (70% in water, 30 µL, 0.23 mmol) was added and the reaction mixture was further stirred for 10 min. Then the reaction mixture was diluted with 20 mL $CHCl_3$, washed once with saturated $NaHCO_3$ (20 mL) and brine (20 mL). The organic layer was dried with $MgSO_4$ and concentrated. The residue was further purified by preparative TLC on silica (v:v CH_2Cl_2/toluene/ isopropanol 85:10:3) to give the desired product (25 mg, 43%). ^1H NMR (300 MHz, $CDCl_3$) δ 8.58 – 8.23 (m, 6H), 7.77 – 7.57 (m, 6H), 7.48 – 7.29 (m, 6H), 4.55 – 3.99 (m, 22H), 2.83 (dq, *J* = 33.3, 5.9 Hz, 16H), 2.11 (s, 6H), 1.85 (d, *J* = 7.7 Hz, 6H), 1.53 (d, *J* = 7.9 Hz, 6H), 1.06 (td, *J* = 7.3, 2.2 Hz, 9H). ^{31}P NMR (121 MHz, $CDCl_3$) δ -2.43. HRMS-NSI (*m/z*): $[M+H]^+$ calcd for $C_{94}H_{83}N_{11}O_{12}P_2$, 1620.5771; found, 1620.5840.

Preparation of compound **8b**

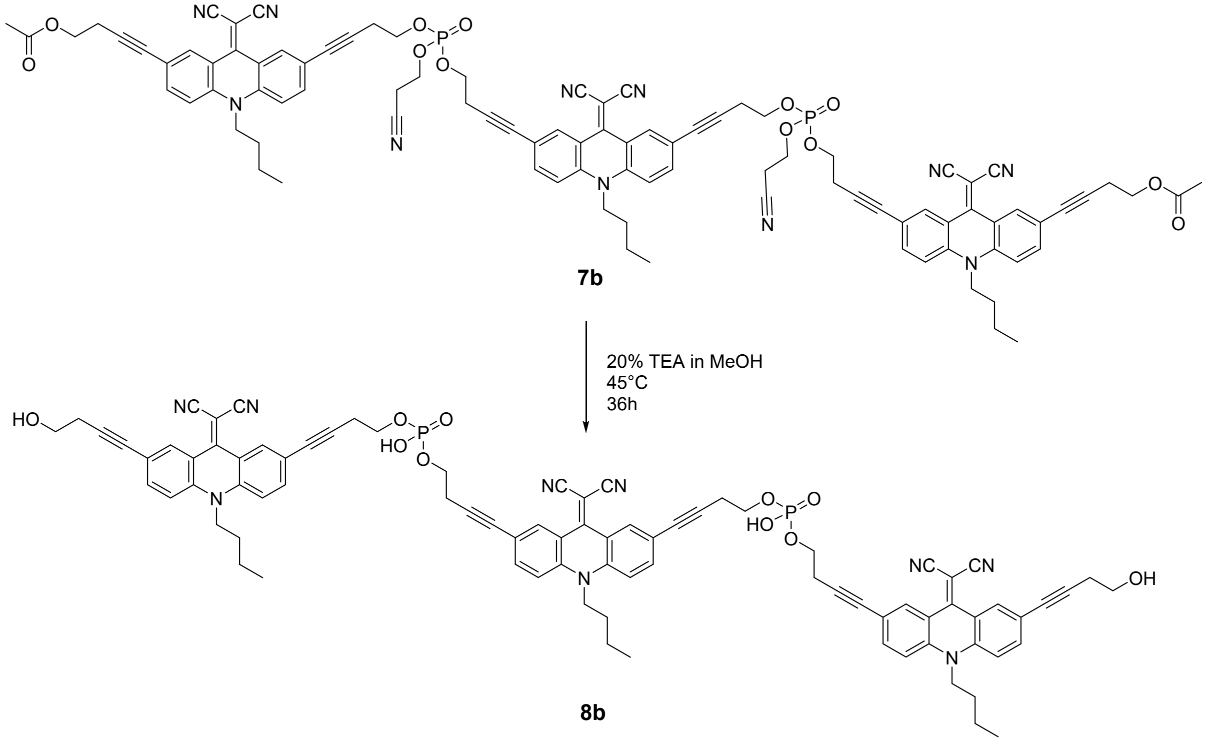

7b (25 mg) was dissolved in a solution of 20% TEA in methanol (20 mL) and stirred at 45°C for 36 h. The sample was further lyophilized and purified by RP-HPLC (Shimadzu LC-20AT, ReproSil 100 C8, 5,0 µm, 250×4 mm) at 40 °C with a flow rate of 1 mL/min. Solvent A: aqueous 2.1 mM TEA / 25 mM 1,1,1,3,3,3-hexafluoropropan-2-ol (HFIP) pH 8; solvent B: acetonitrile; gradient: B [%] tR [min]: 15 (0), 15 (2), 60 (20). The purified trimer was dissolved in EtOH and the absorbance of the compound was measured to determine the concentration of the stock solution. The Beer-Lambert law was applied to determine the concentrations. The molar absorption coefficient was determined to be: ε(8b, 504 nm): 44'200 $L\cdot mol^{-1}\cdot cm^{-1}$. The final product is a red solid (10 mg, 46%). HRMS-NSI (*m/z*): $[M-2H]^{2-}$ calcd for $C_{84}H_{71}O_{10}N_9P_2$, 713.7405; found, 713.7385.

2.2. NMR Spectra

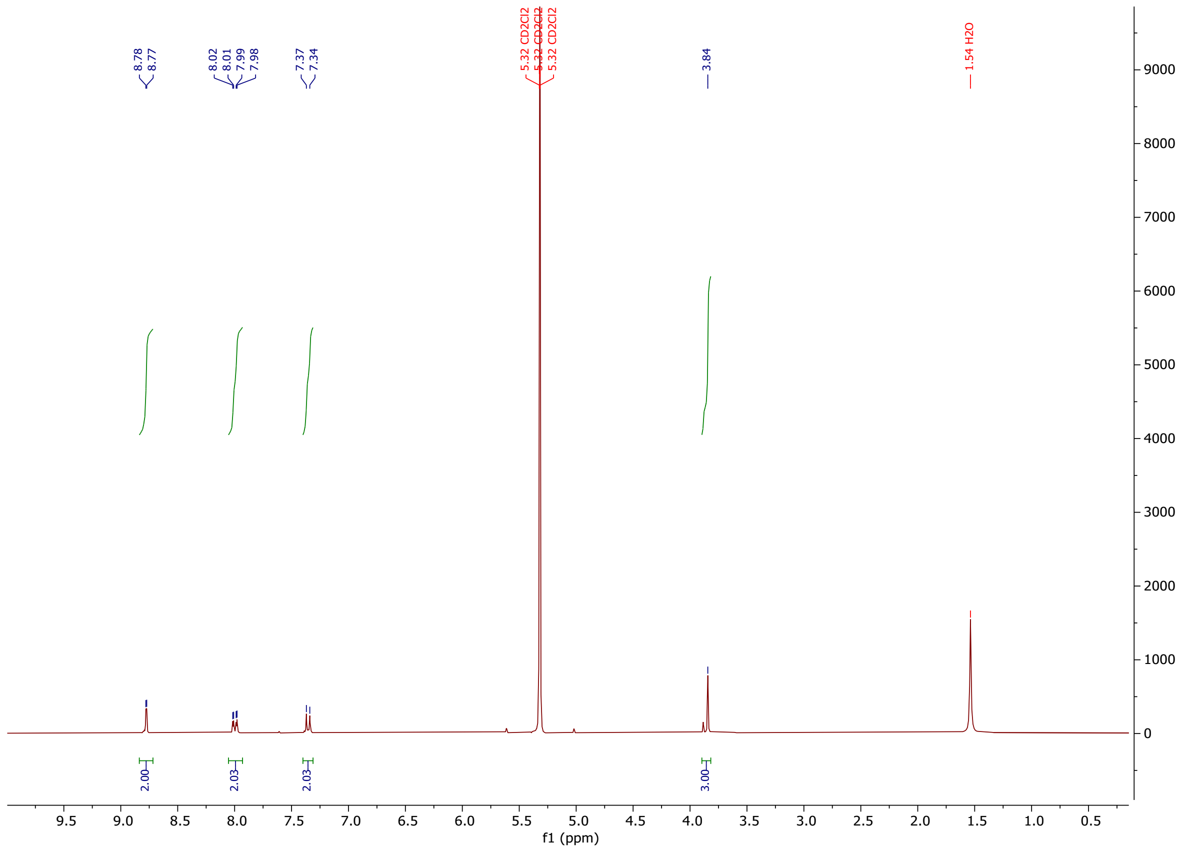

Figure 1 ^{1}H NMR spectra of 2a

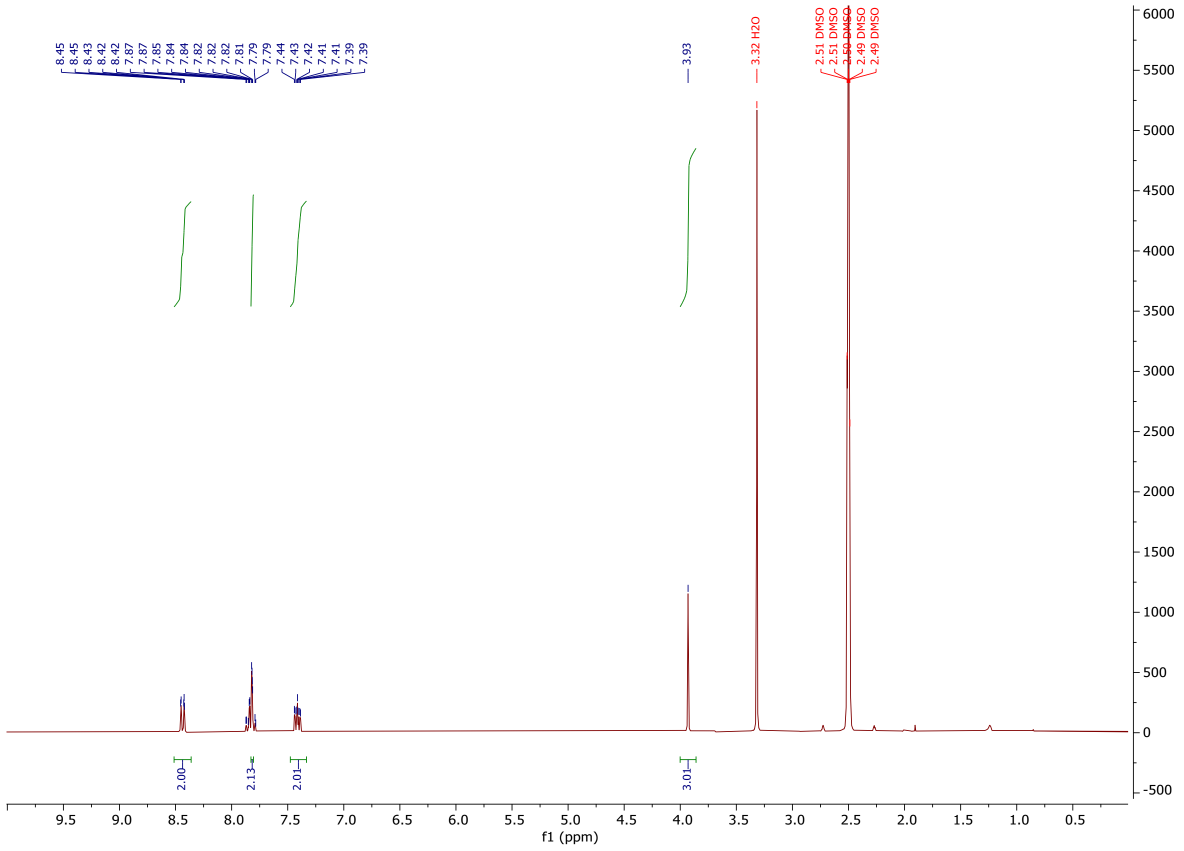

Figure 2 ^{1}H NMR spectra of 3a

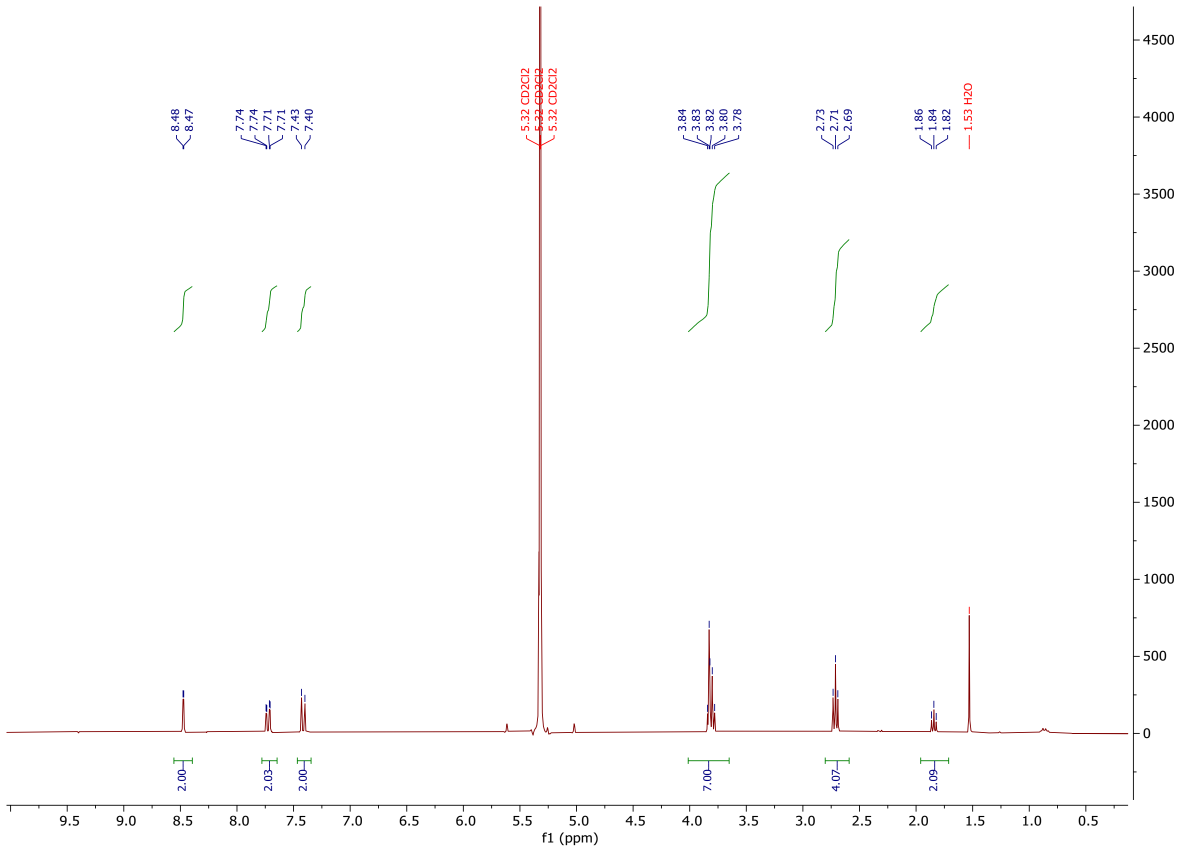

Figure 3 ^{1}H NMR spectra of 4a

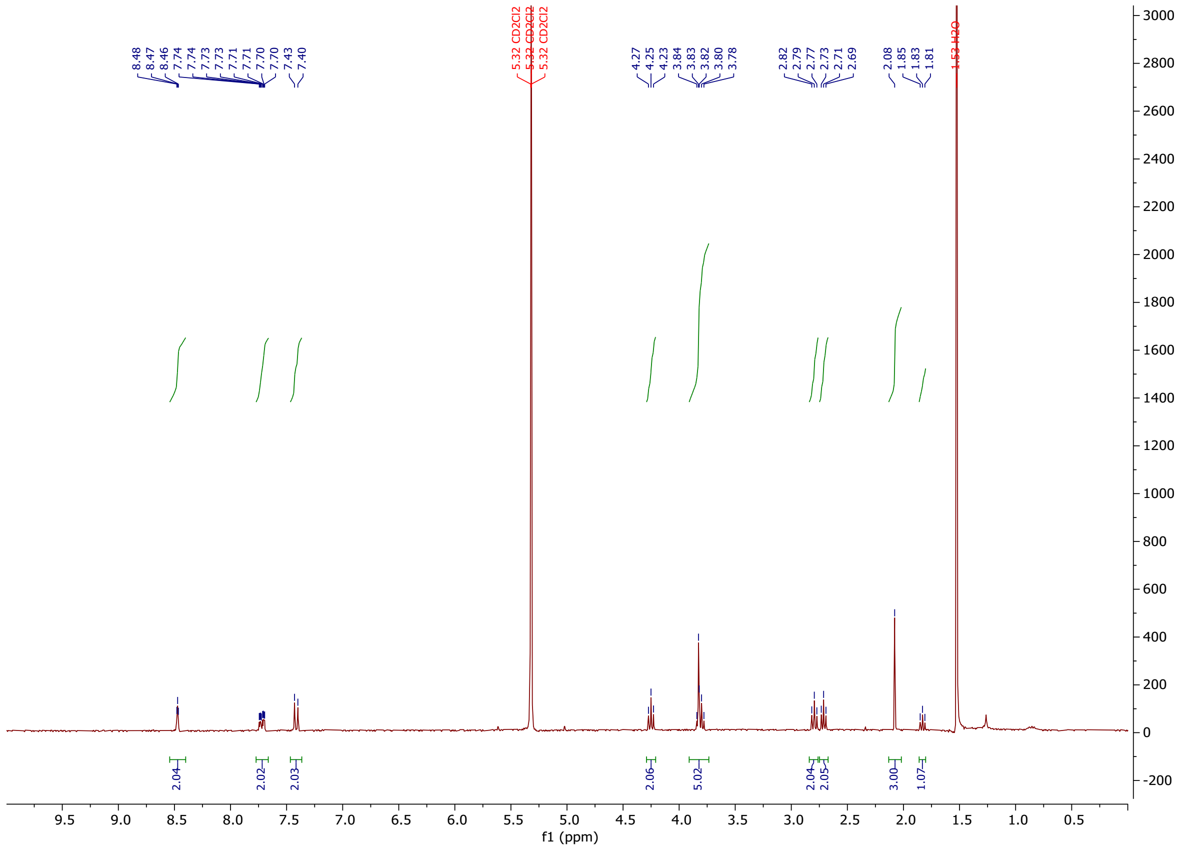

Figure 4 ^{1}H NMR spectra of 5a

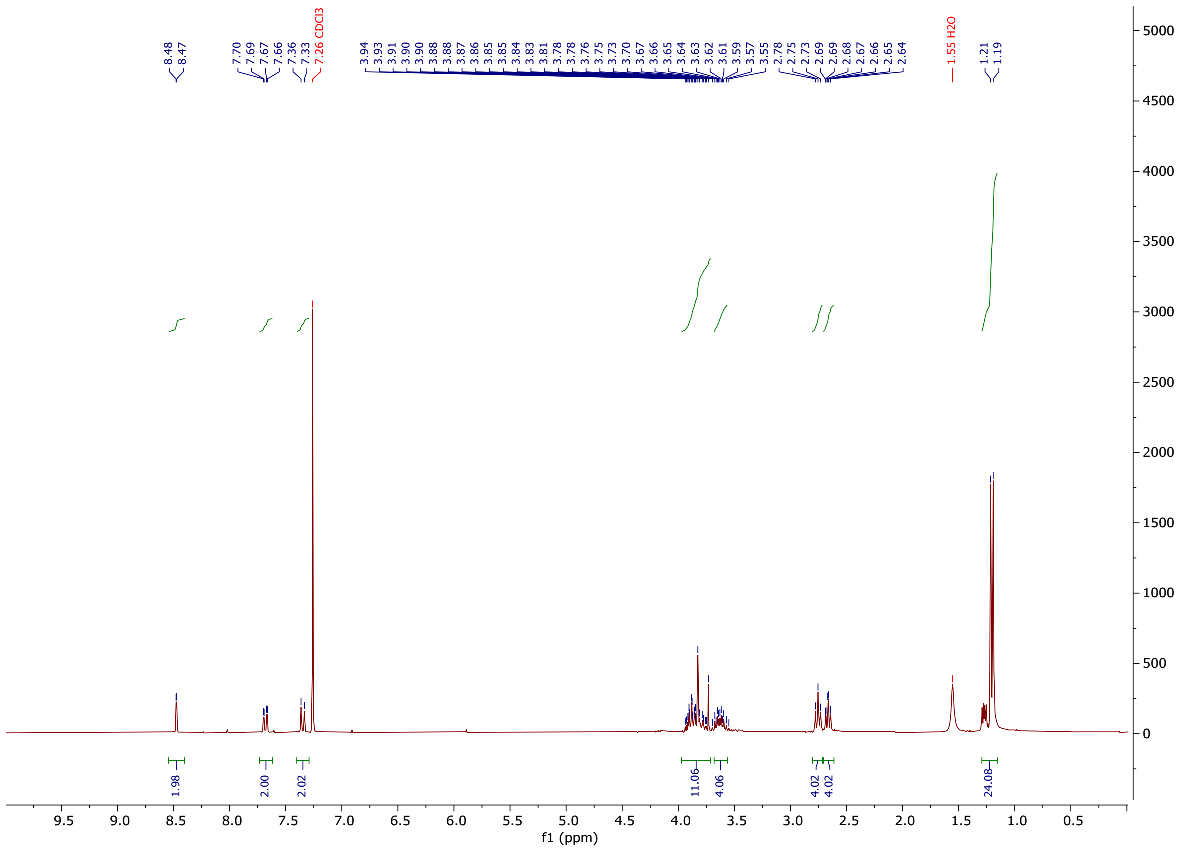

Figure 5 ^{1}H NMR spectra of 6a

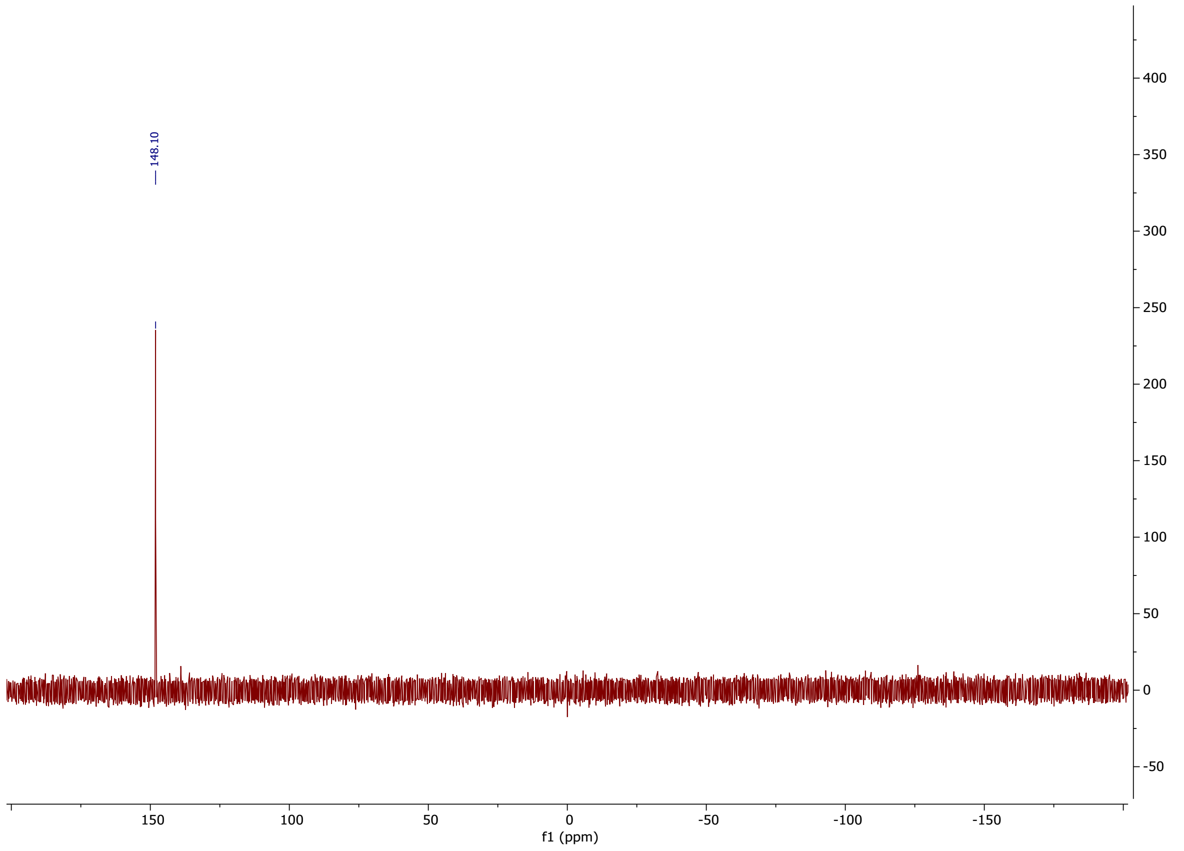

Figure 6 ^{31}P NMR spectra of 6a

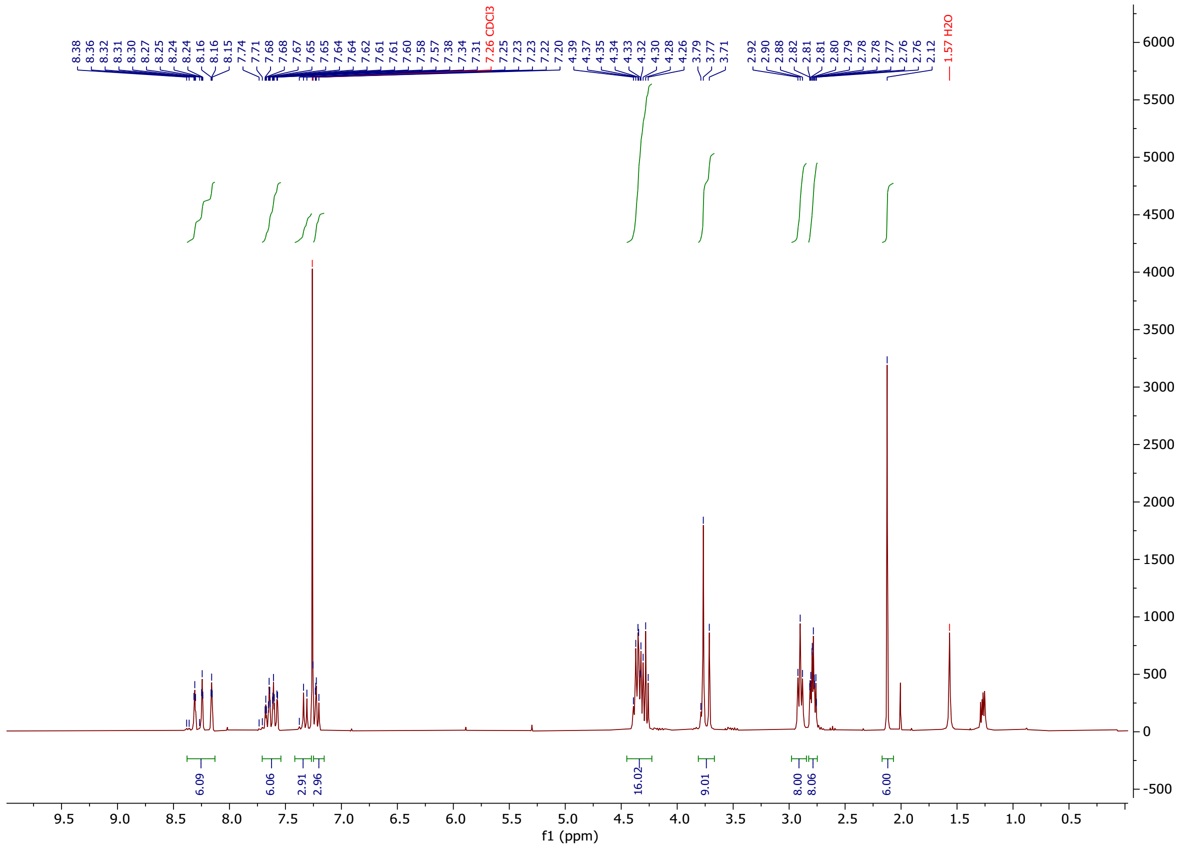

Figure 7 ^{1}H NMR spectra of 7a

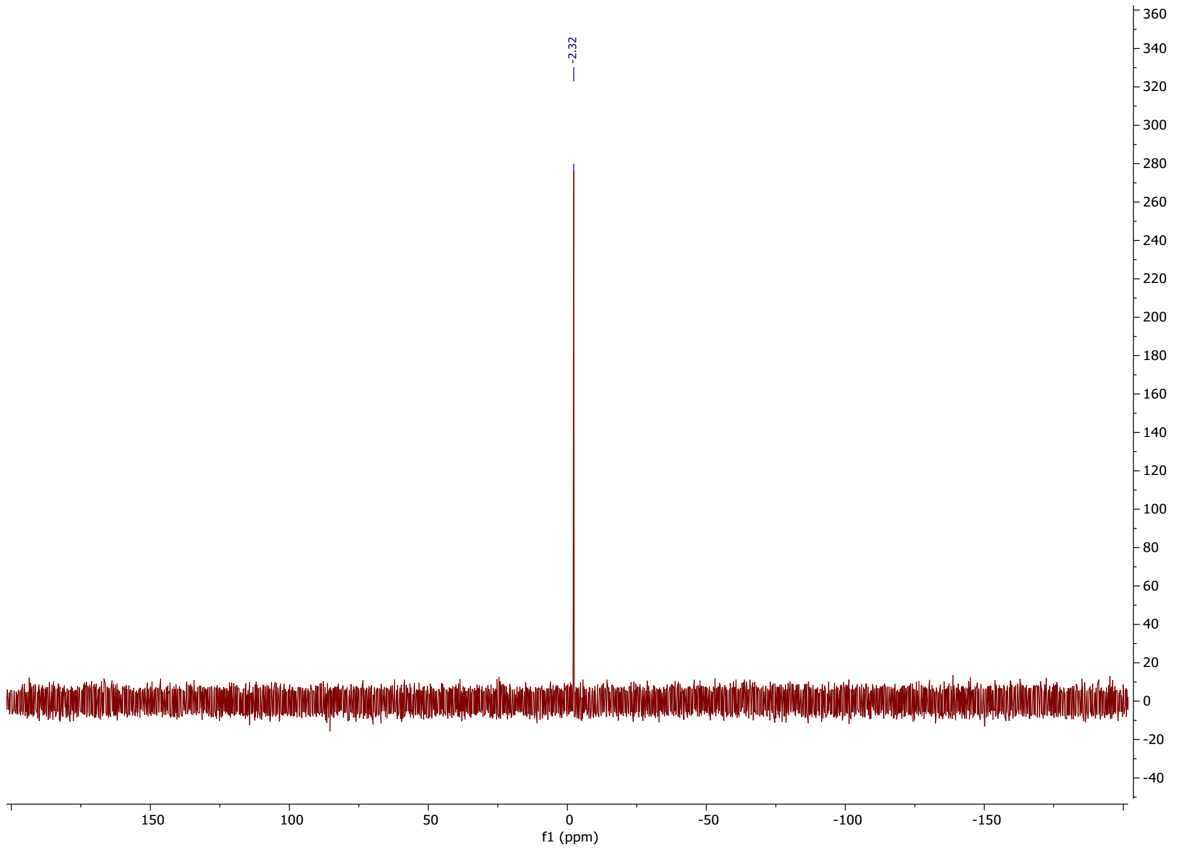

Figure 8 ^{31}P NMR spectra 7a

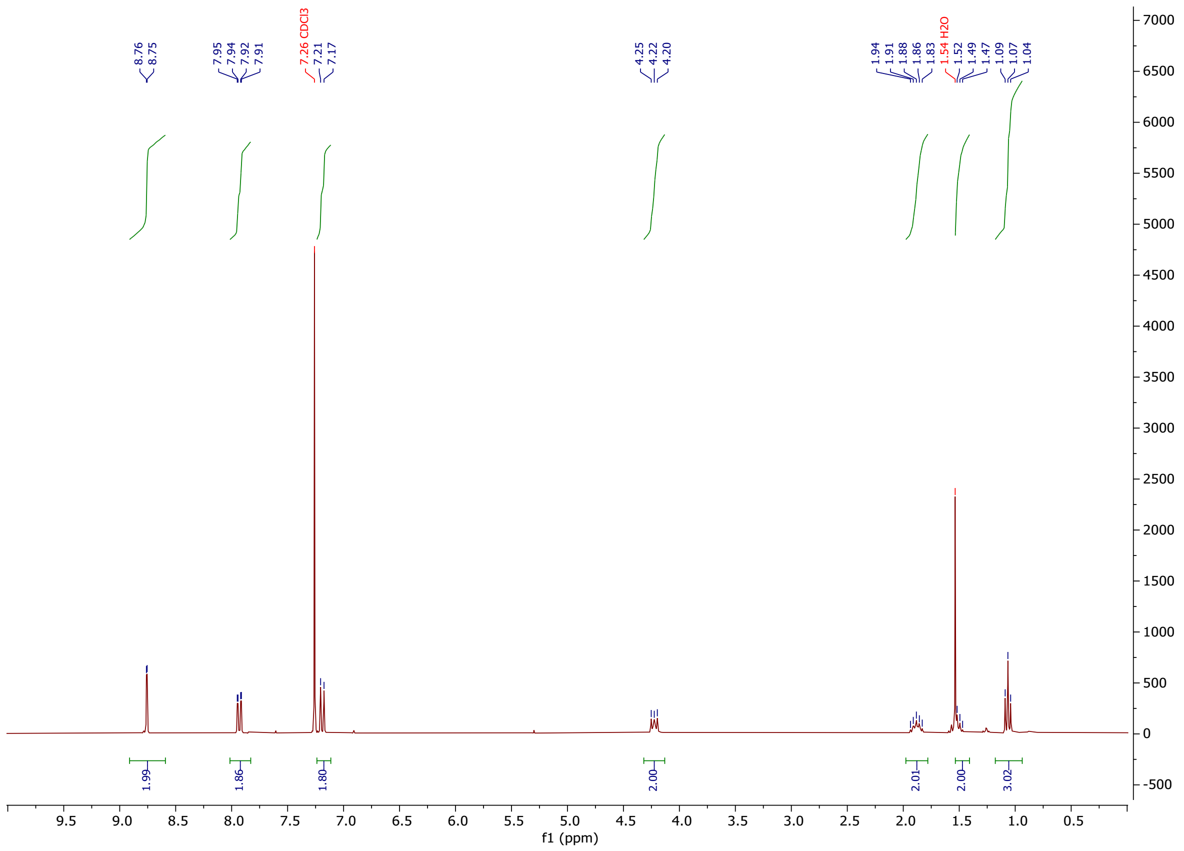

Figure 9 ^{1}H NMR spectra of 3b

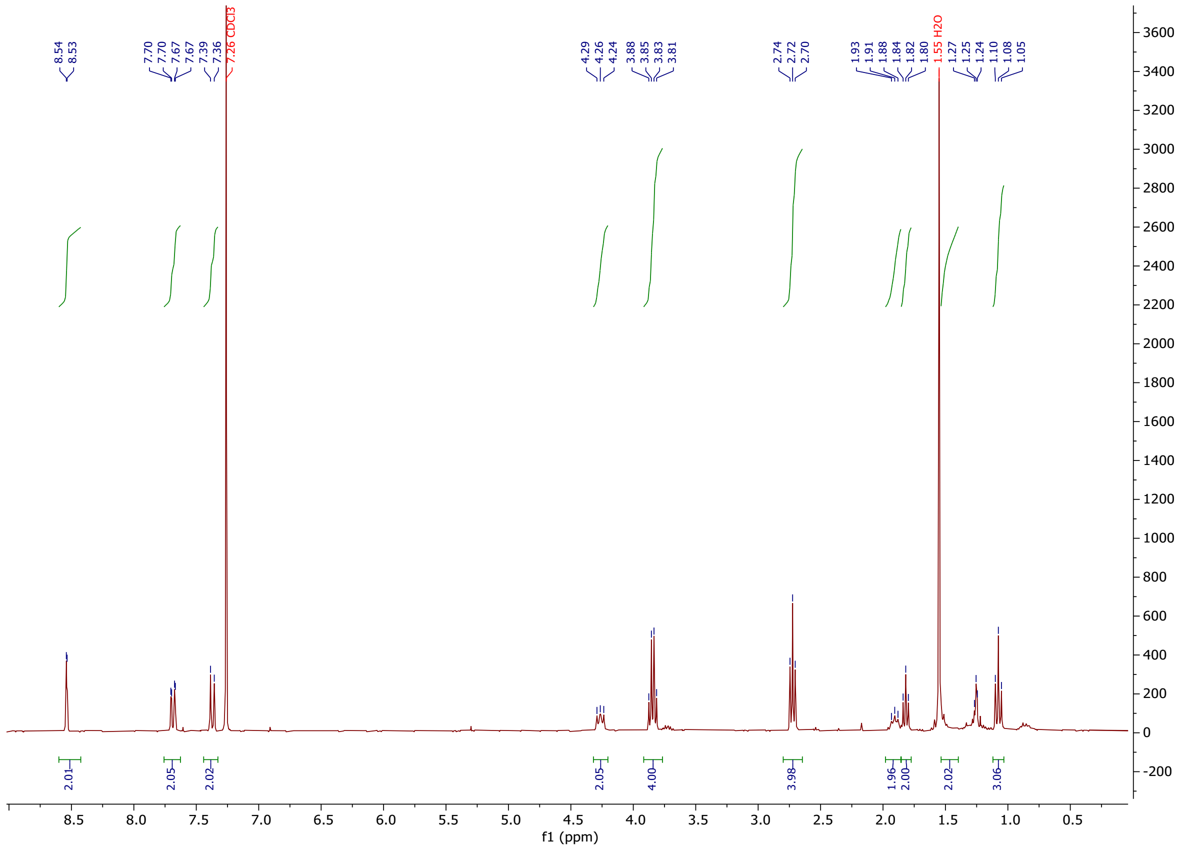

Figure 10 ^{1}H NMR spectra of 4b

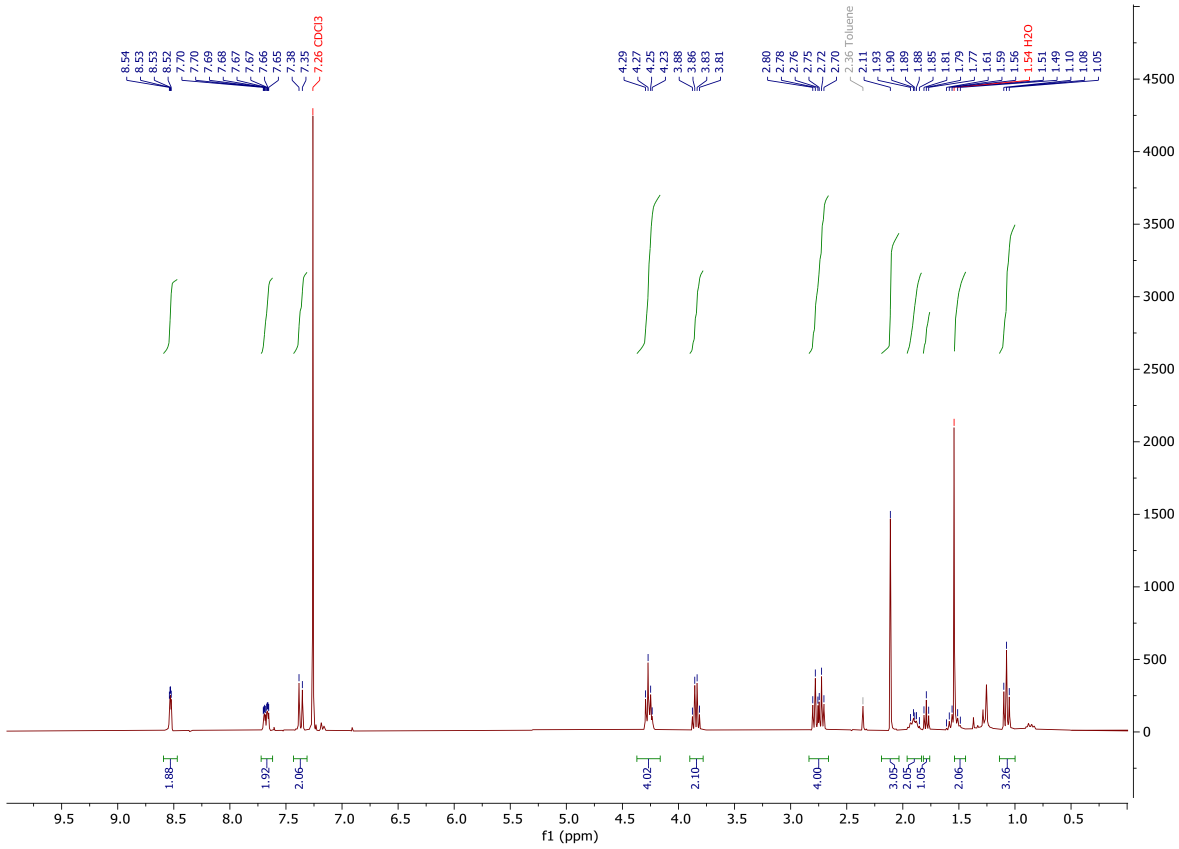

Figure 11 ^{1}H NMR spectra of 5b

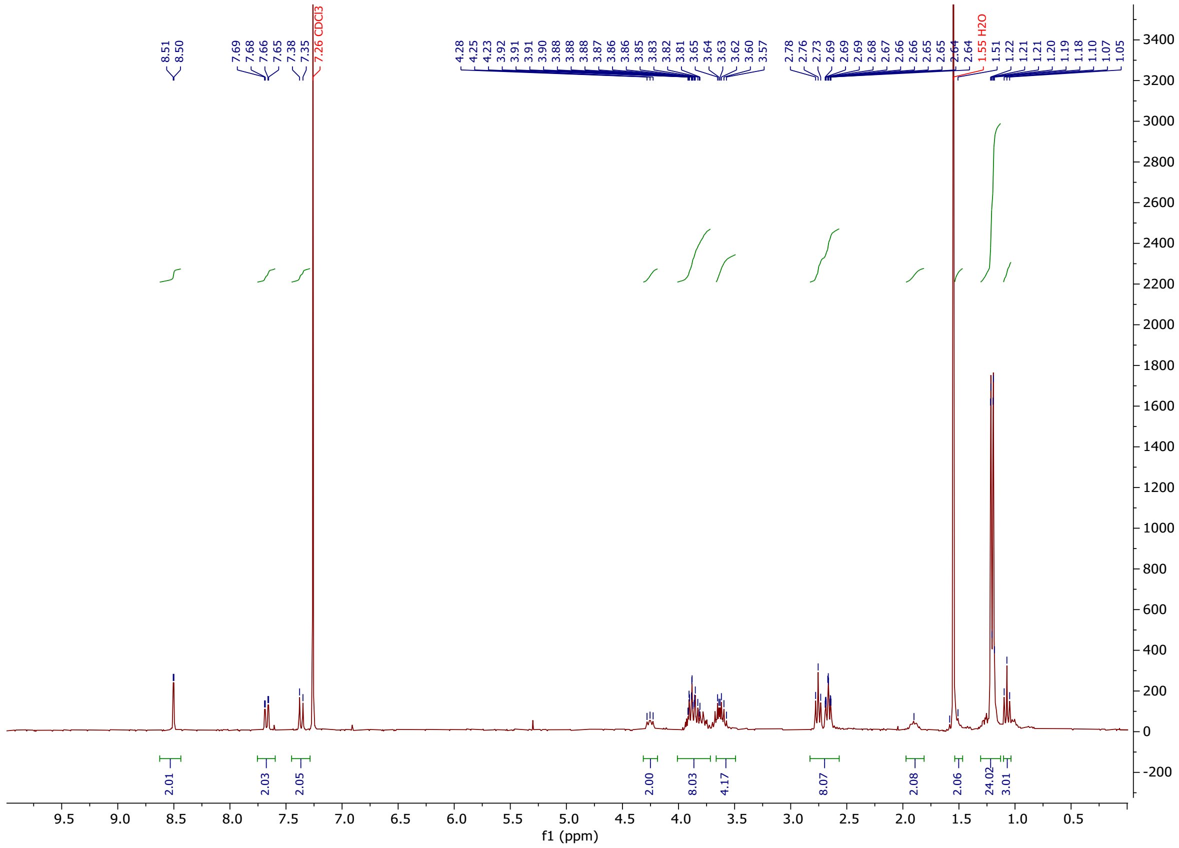

Figure 12 ^{1}H NMR spectra of 6b

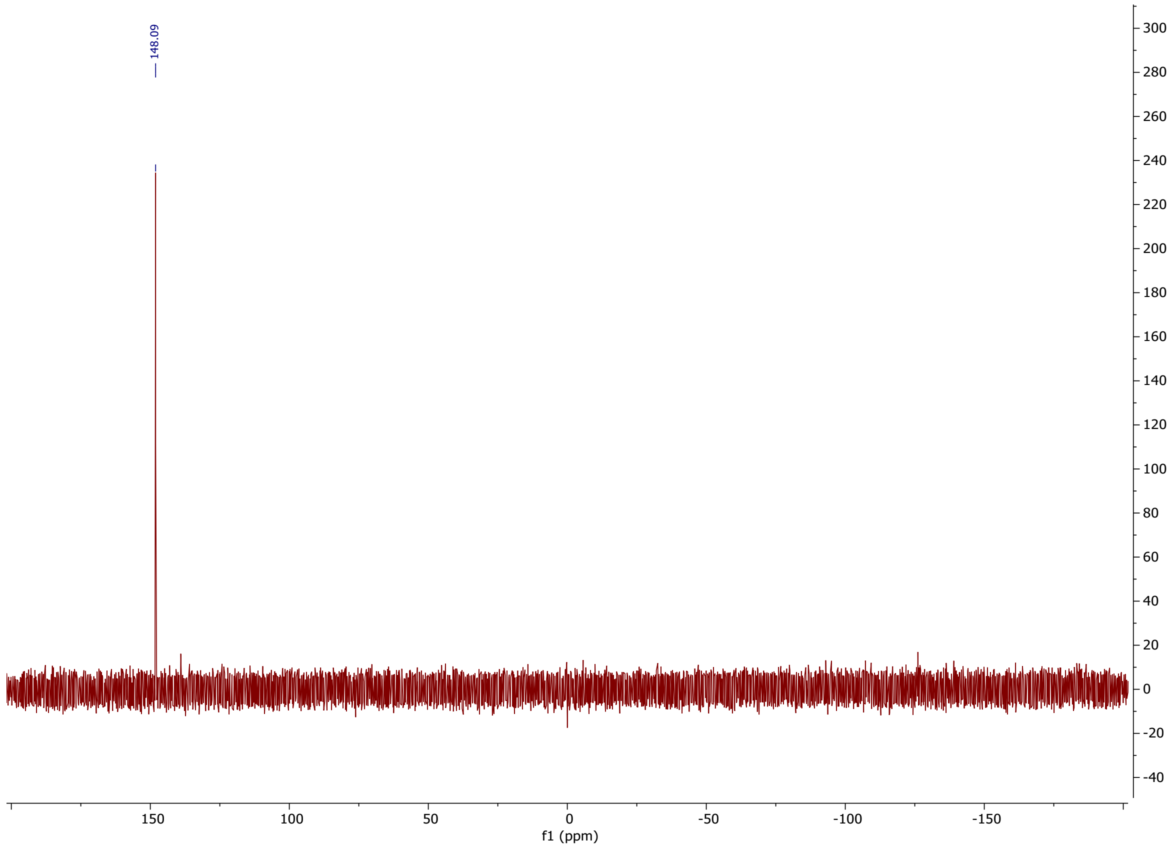

Figure 13 ^{31}P NMR spectra of 6b

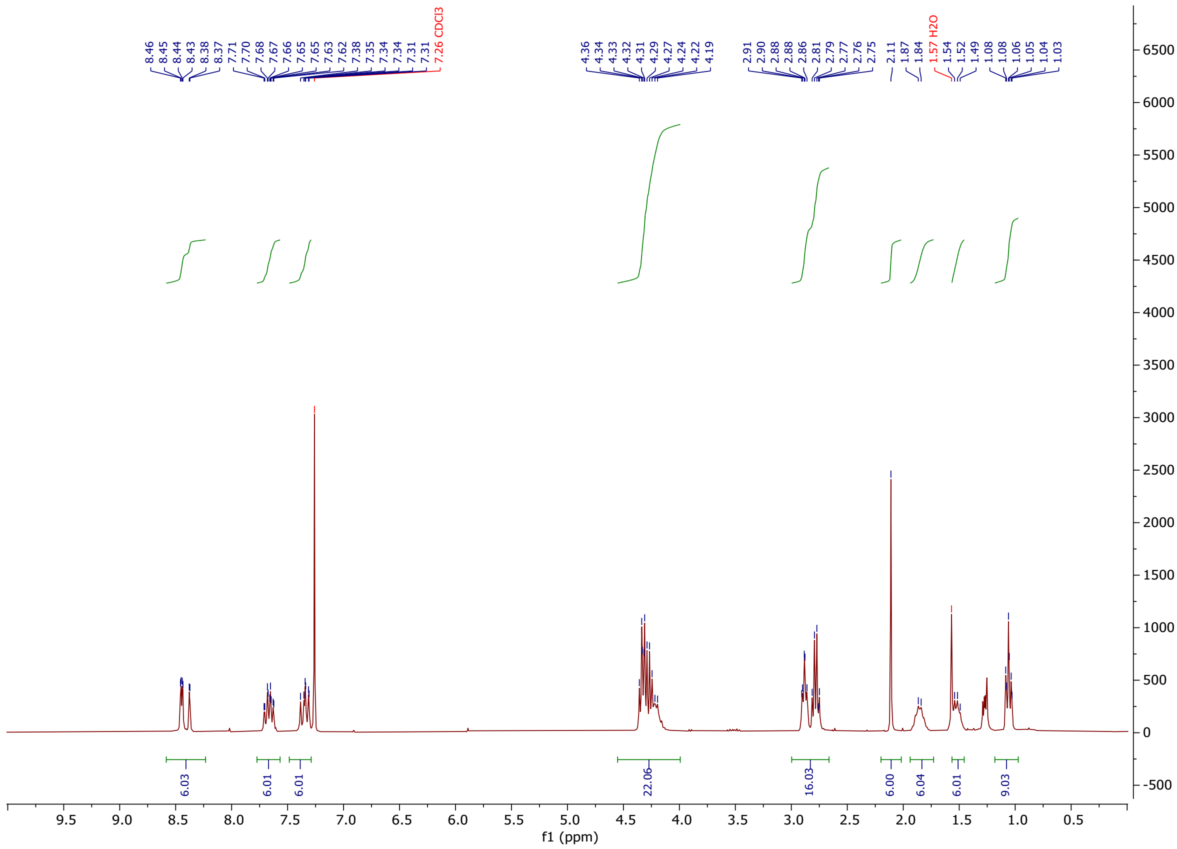

Figure 14 ^{1}H NMR spectra of 7b

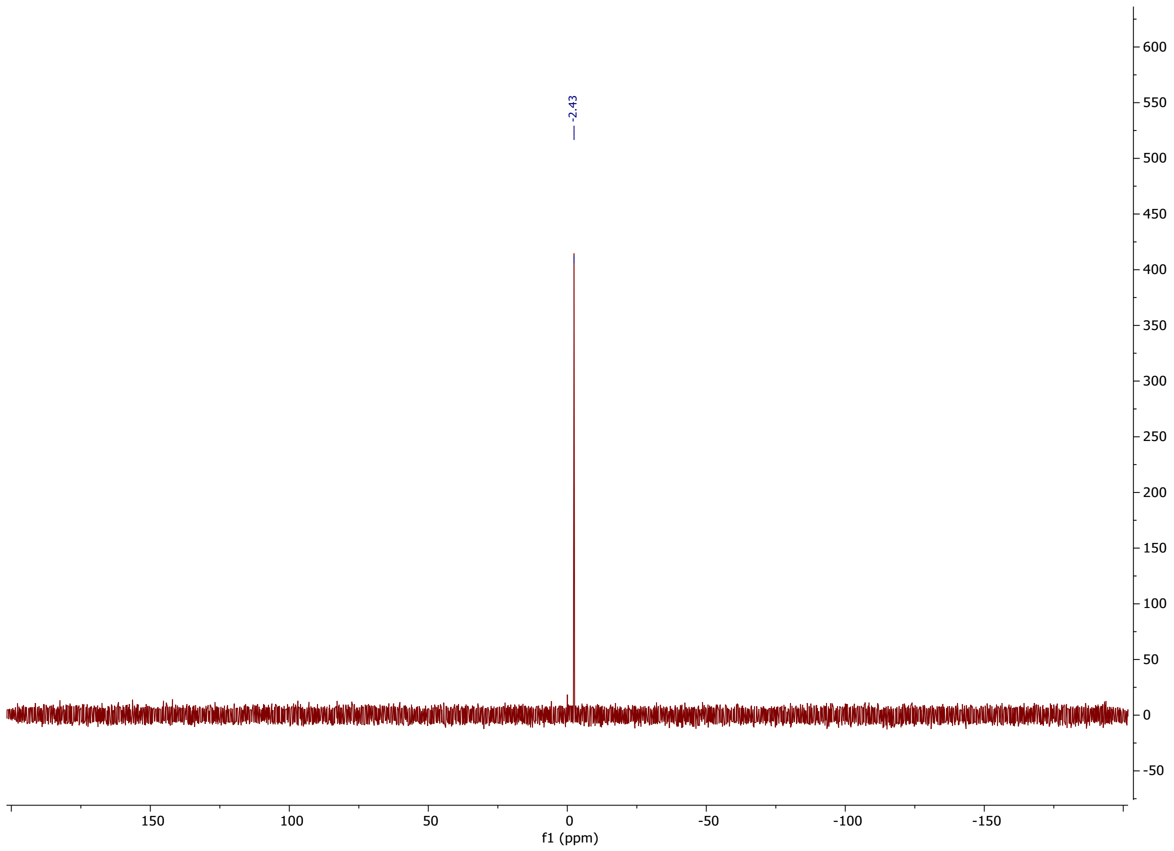

Figure 15 ^{31}P NMR spectra of 7b

2.3. HPLC traces

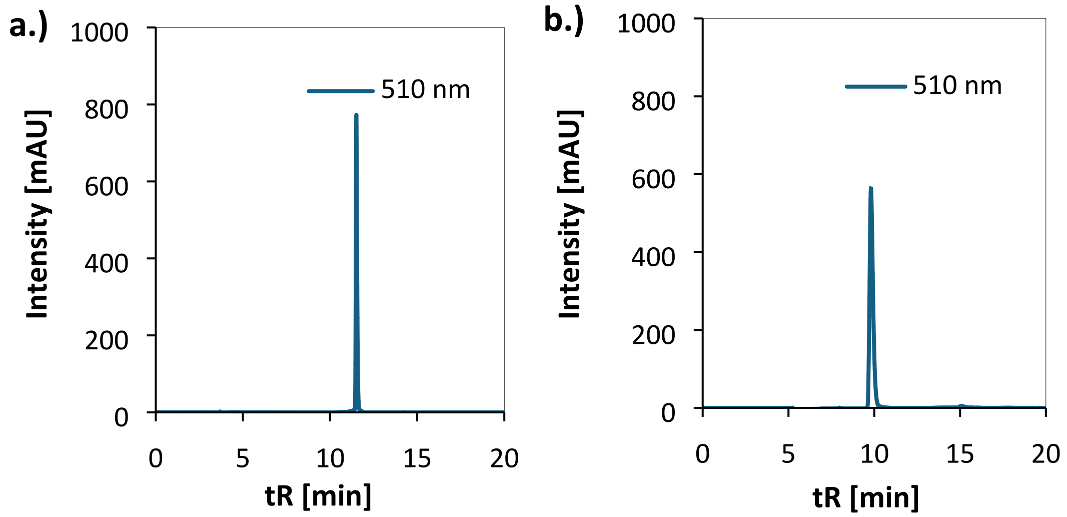

Figure 16 HPLC traces of a.) 8a, t_R = 11.5 min; b.) 8b, t_R = 9.8 min

2.4. MS Spectra

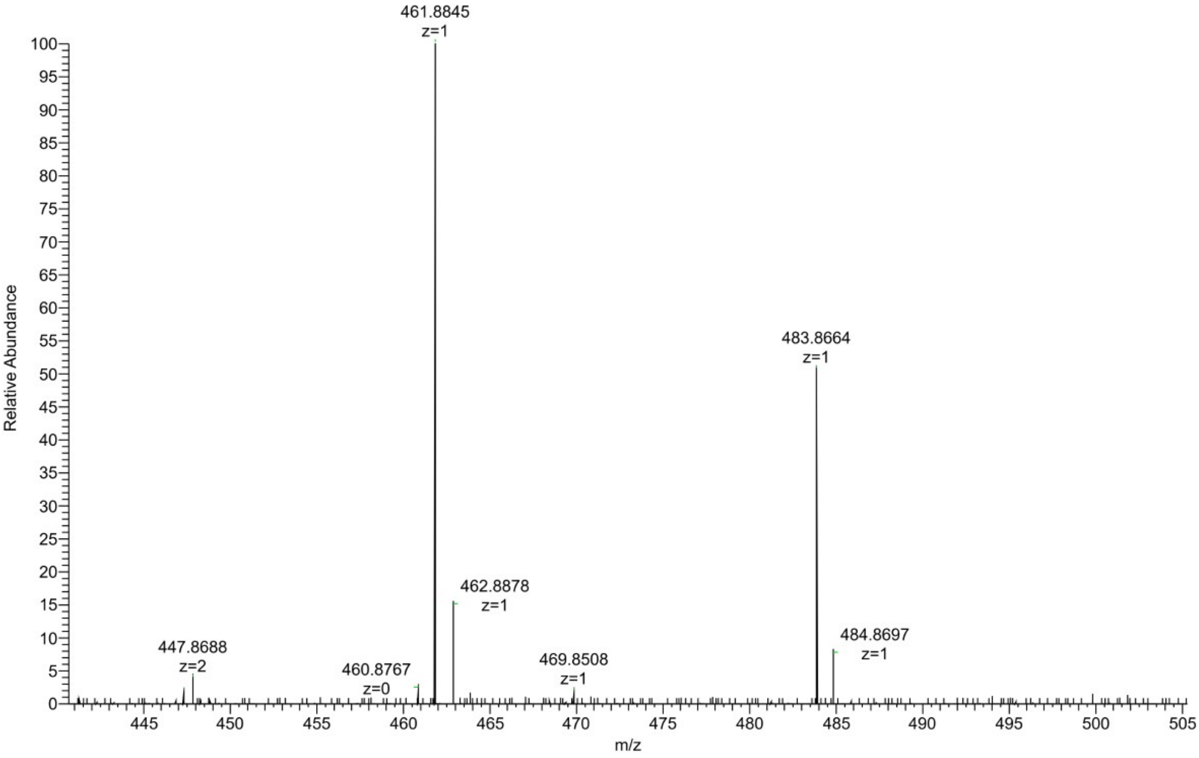

Figure 17 HR-MS of 2a in the presence of Na⁺ adduct

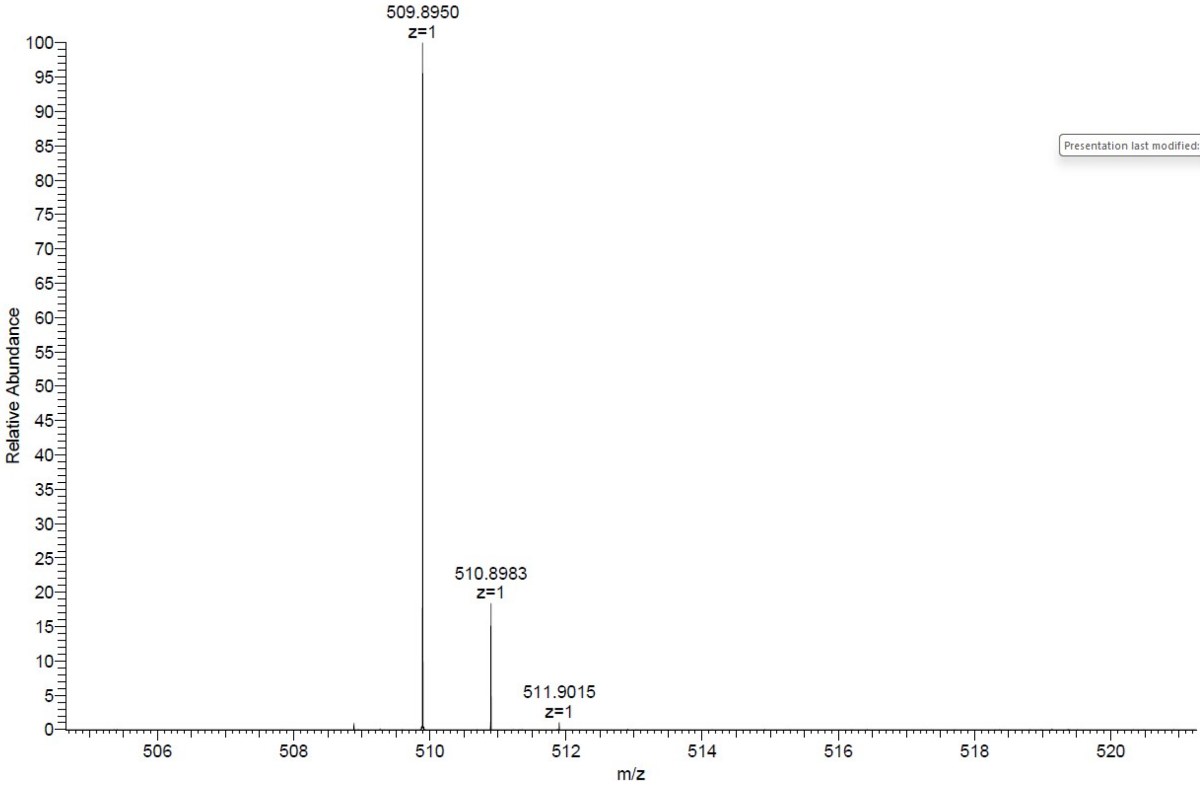

Figure 18 HR-MS of 3a

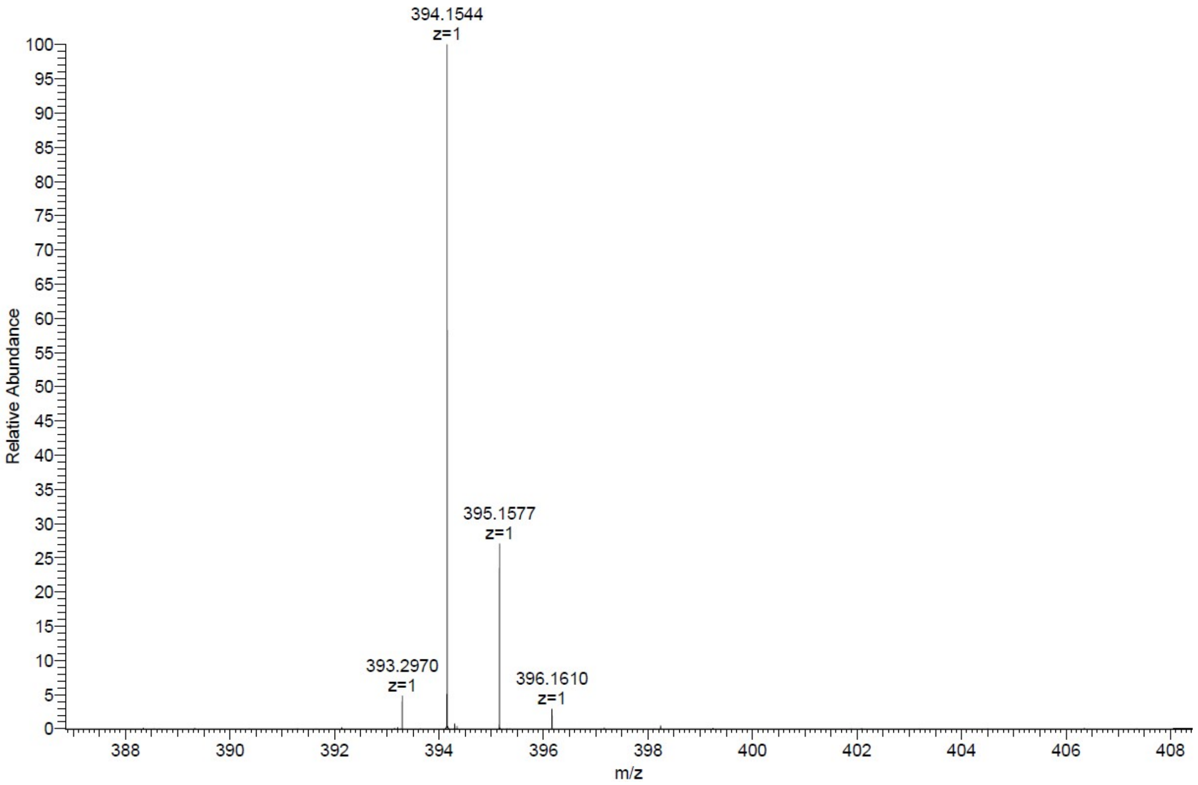

Figure 19 HR-MS of 4a

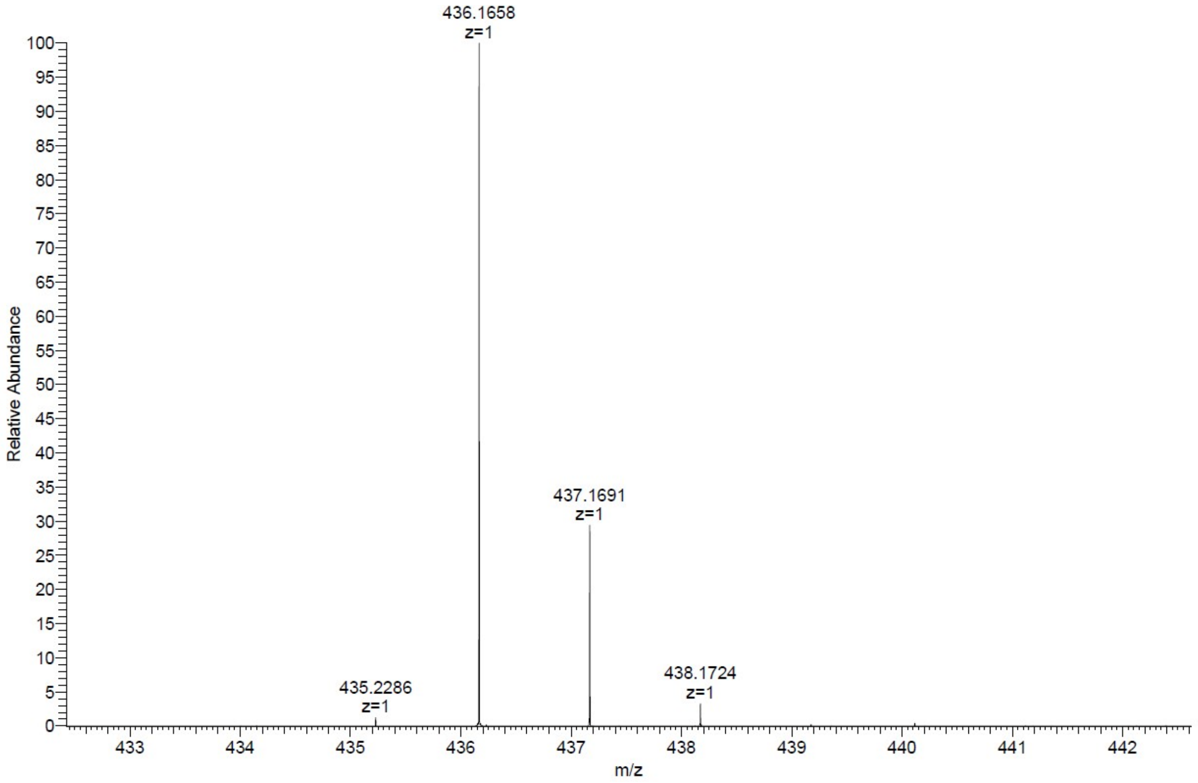

Figure 20 HR-MS of 5a

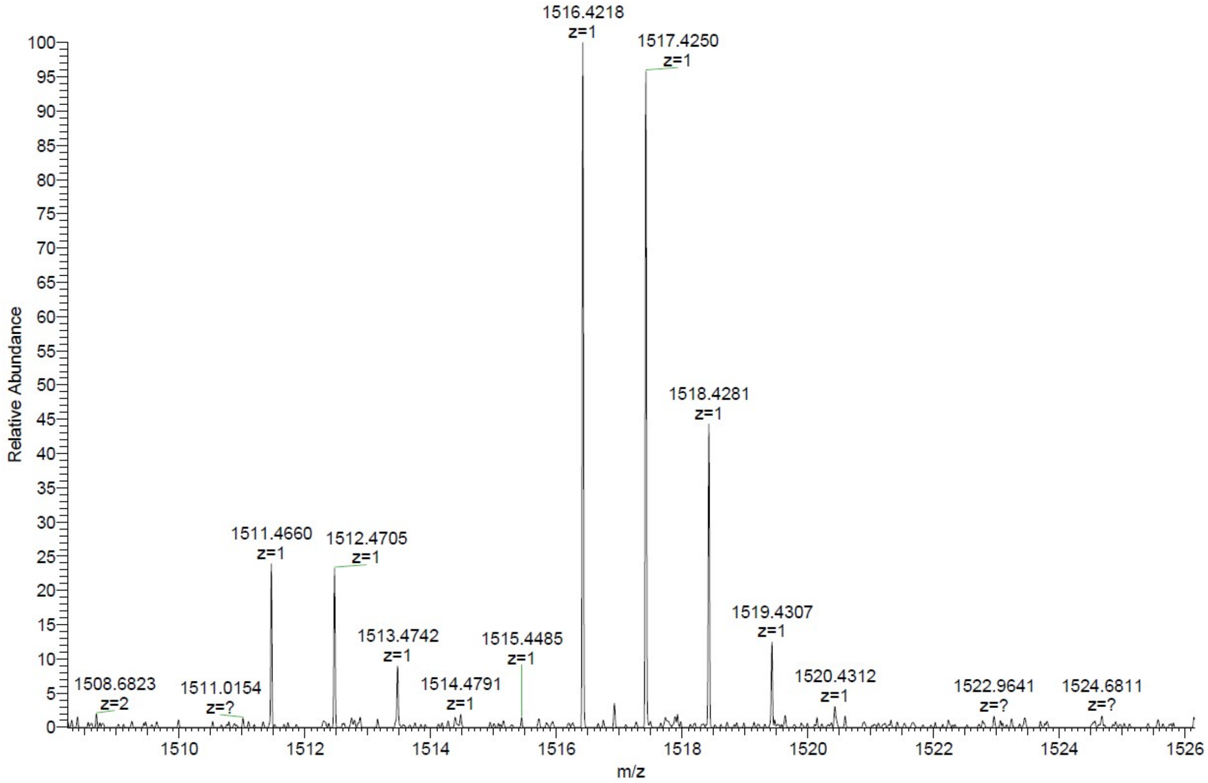

Figure 21 HR-MS of 7a in the presence of Na^{+} and NH_4^{+} adducts

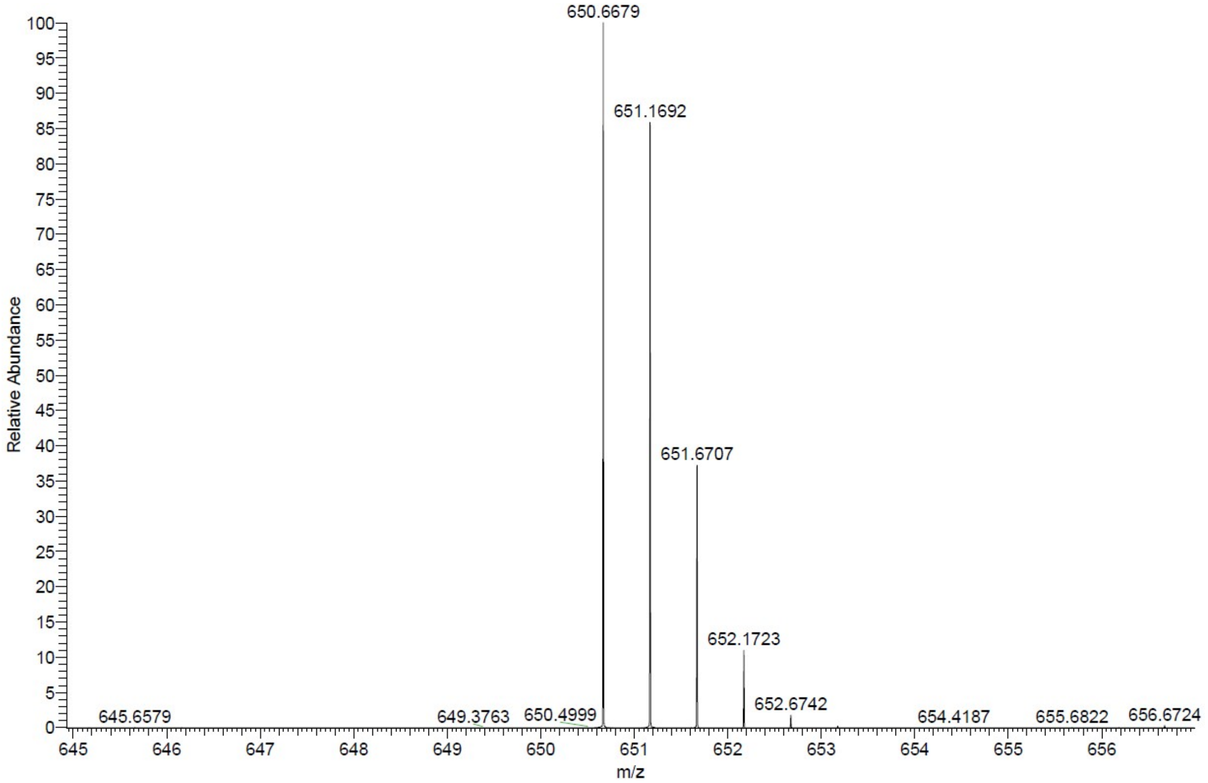

Figure 22 HR-MS of 8a

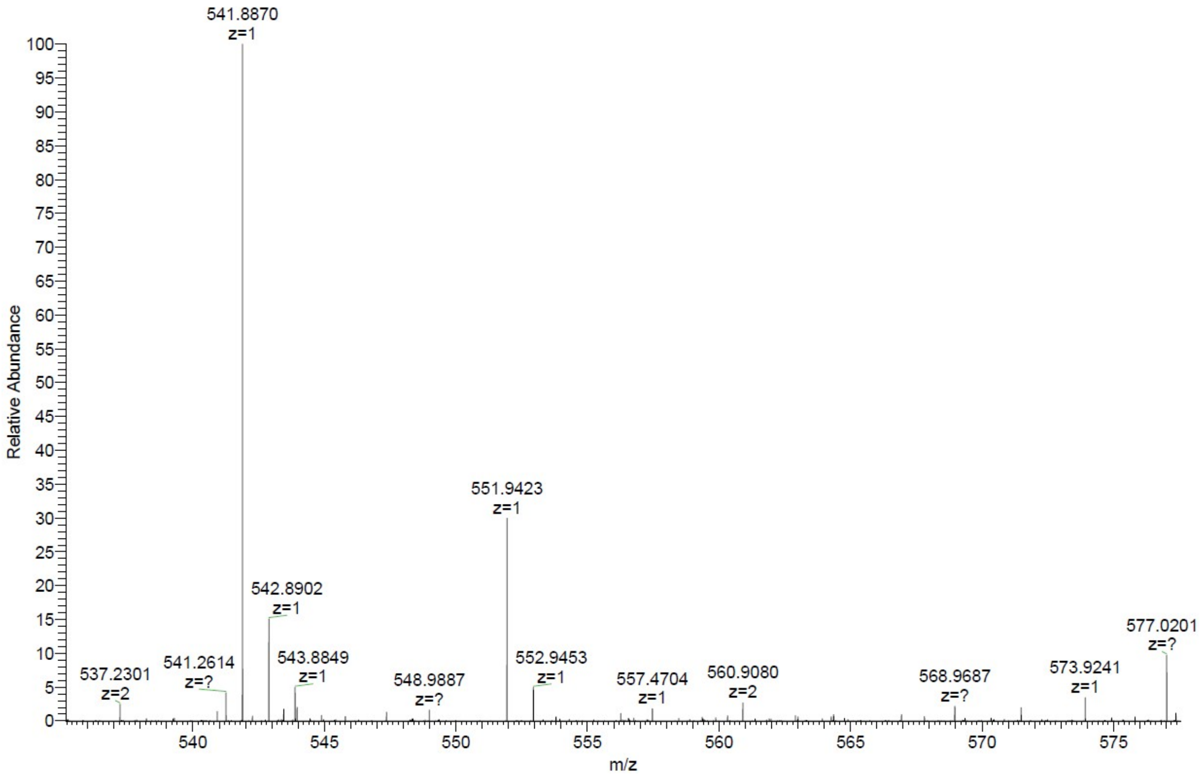

Figure 23 HR-MS of 3b

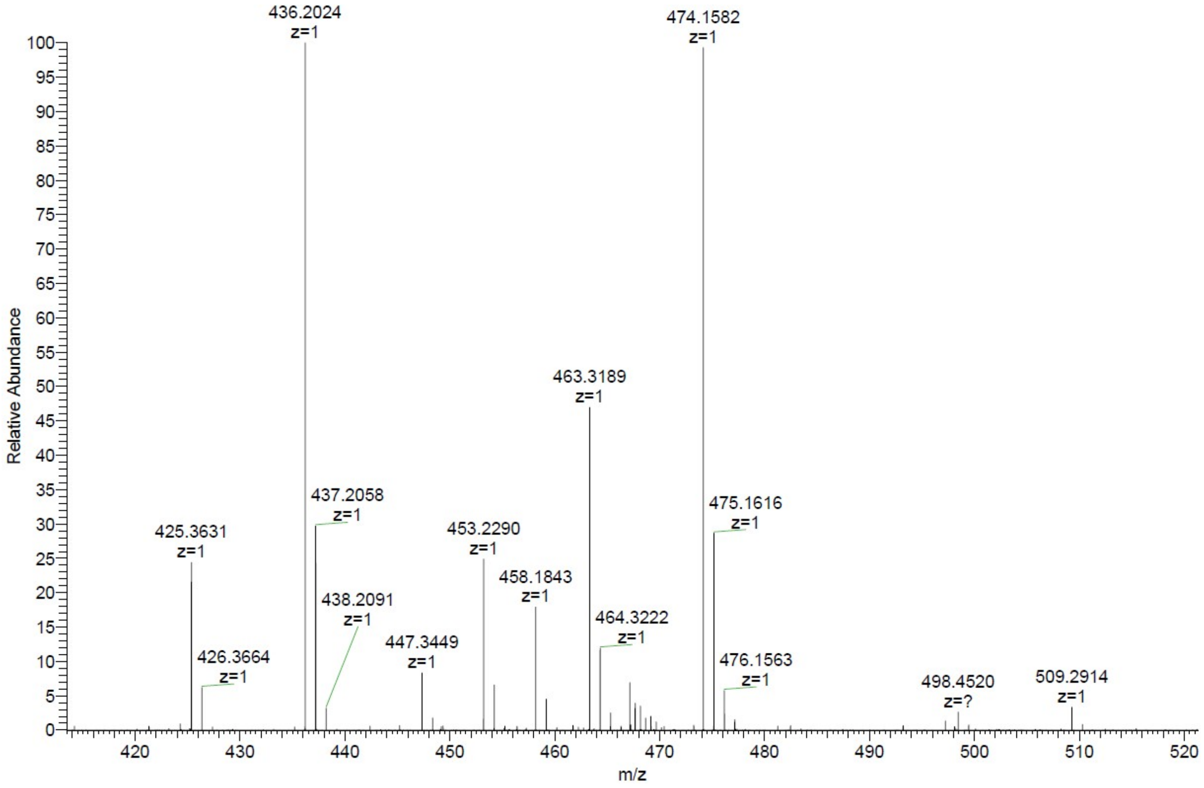

Figure 24 HR-MS of 4b in the presence of a K$^+$ adduct

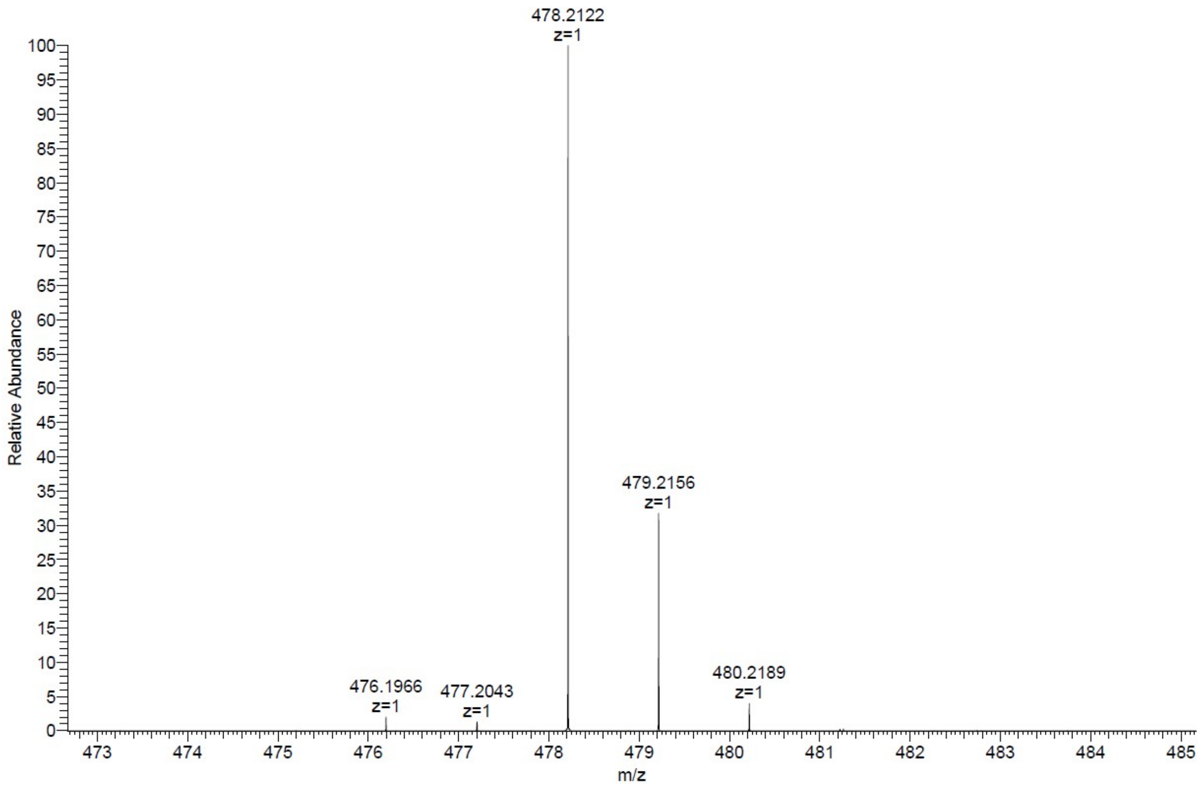

Figure 25 HR-MS of 5b

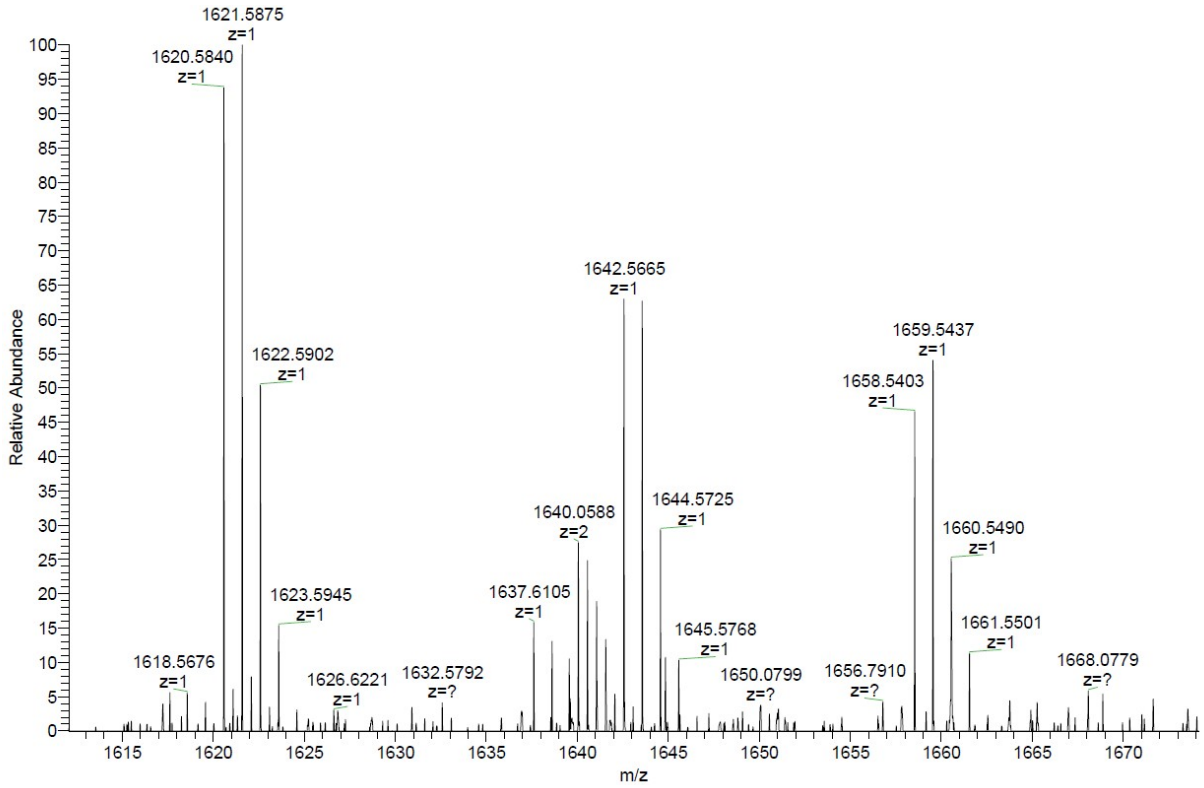

Figure 26 HR-MS of 7b in the presence of Na^+ and K^+ adducts

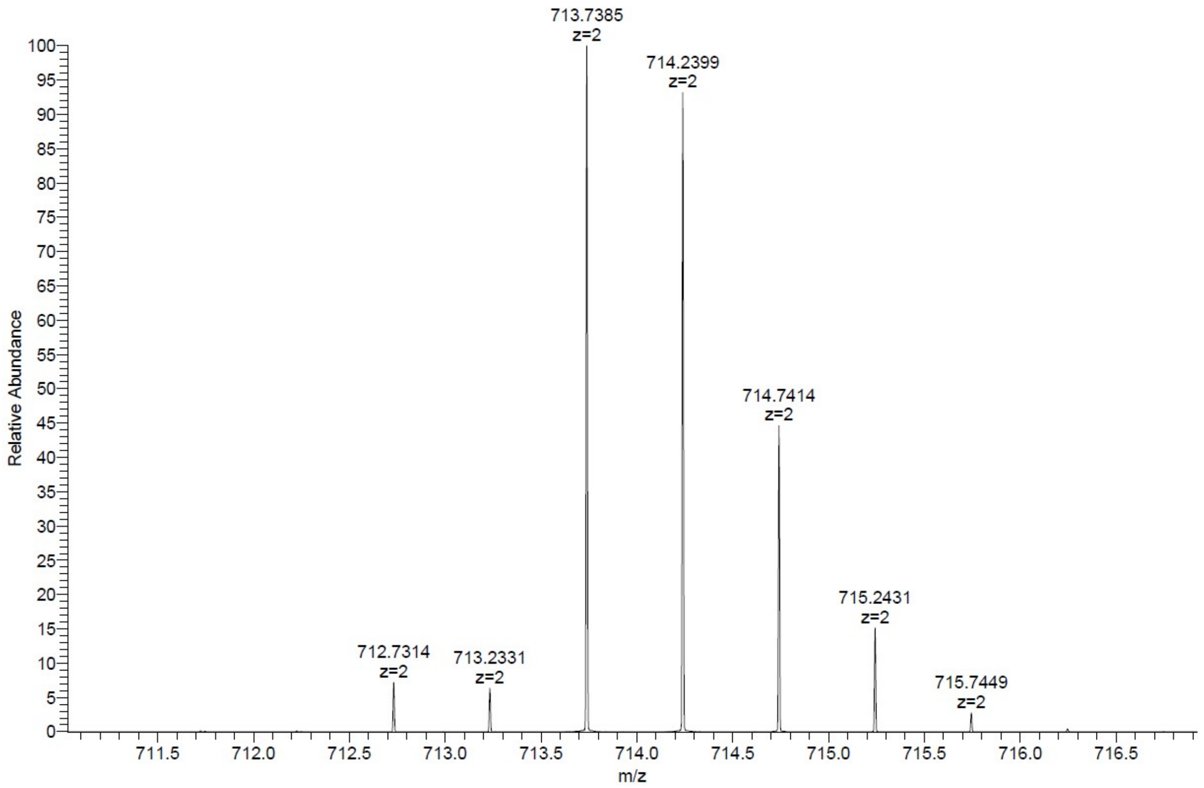

Figure 27 HR-MS of 8b

3. Preparation of the sample

In an Eppendorf was added subsequently Milli-Q H_2O, EtOH, 100 µL of a sodium phosphate buffer (0.1 M, pH 7.2), NaCl (2 M) and finally the oligomer for a final volume of 1 mL. The solution was then vortexed to ensure a thorough mixing before being transferred to a quartz cuvette. Conditions of all spectroscopic measurements if not indicated differently: Aqueous solution with 3 µM trimer, 10 mM sodium phosphate buffer pH 7.2, 100 mM NaCl, 10 vol% ethanol. The sample solution was heated to 75 °C, then cooled with a gradient of 0.5 °C/min to 20 °C.

4. Excitation Spectra

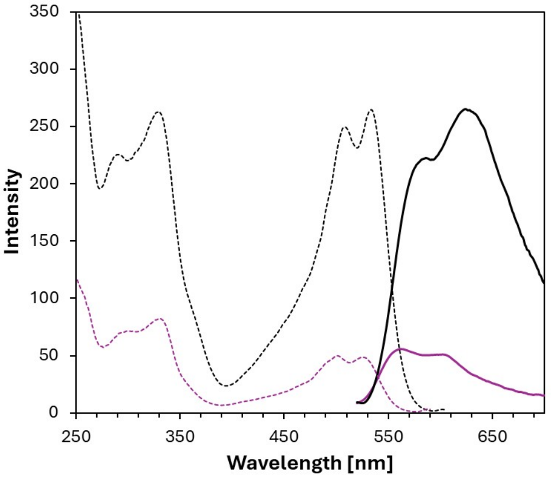

Figure 28 Excitation (dashed) and emission (line) spectra of **8a** (black) and **8b** (pink); λ_{ex} 505 nm; Measurements were performed at 20°C.

5. UV-vis & fluorescence spectra

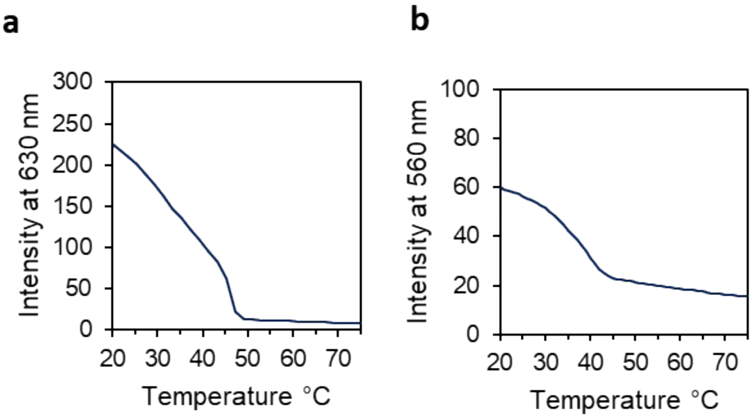

Figure 29 Fluorescence intensity at 630 nm for **8a** (a) and 560 nm for **8b** (b) during cooling (0.5°C/min) from 75 °C to 20 °C. λ_{ex} 505 nm.

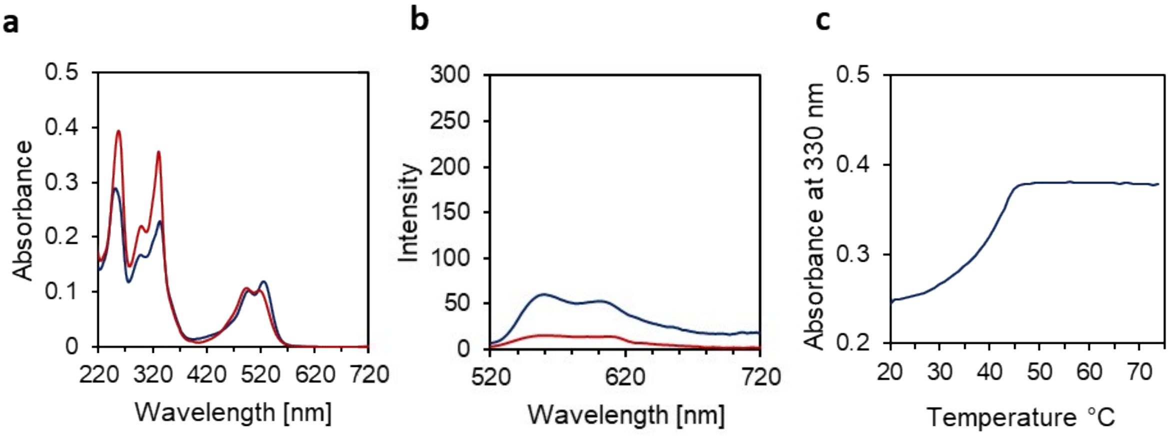

Figure 30 (a,b) UV-vis and fluorescence spectra at 75°C (red) and at 20°C (blue) of **8b**, λ_{ex} 505 nm. (c) Absorbance at 330 nm during cooling (0.5 °C/min) from 75 °C to 20 °C.

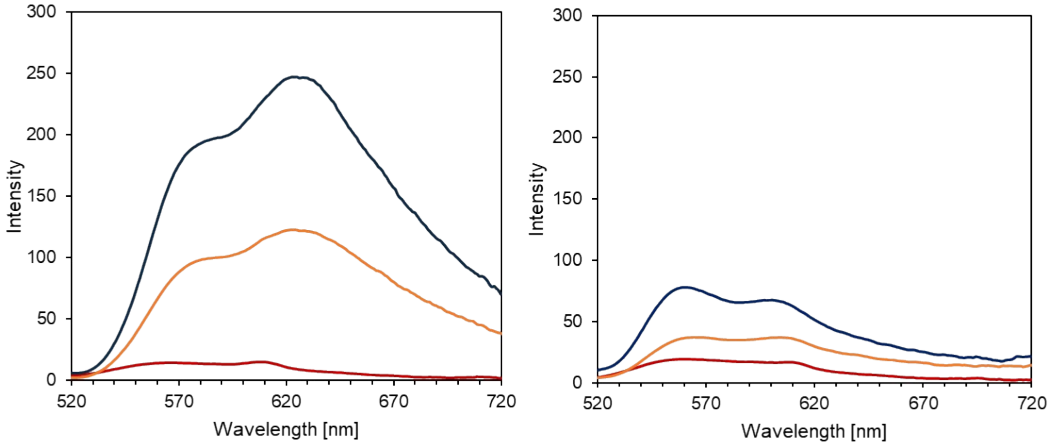

Figure 31 Emission spectra of **8a** (left) and **8b** (right), λ_{ex} 505 nm. After pipetting the samples at 20 °C without a controlled heating/cooling cycle (orange), 75 °C (red), 20 °C (blue).

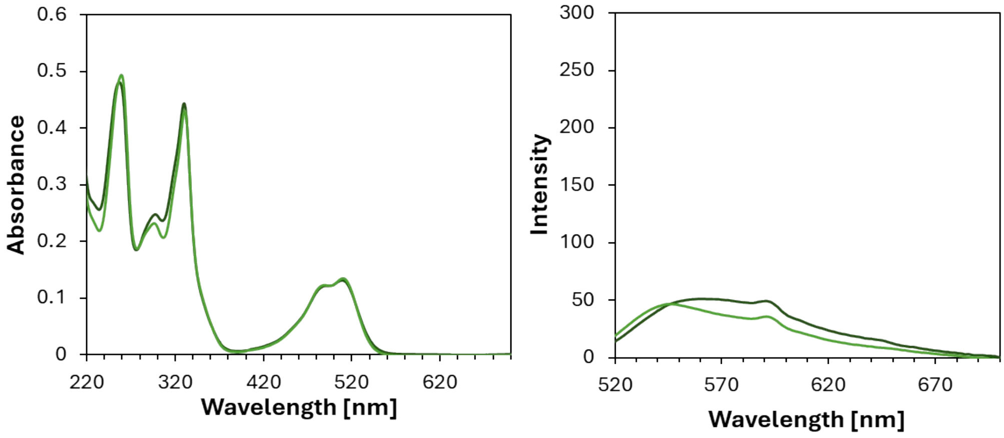

Figure 32 UV-vis (left) and emission spectra of **8a** (dark green) and **8b** (light green), λ_{ex} 505 nm. Conditions: 3 uM trimer in ethanol at room temperature.

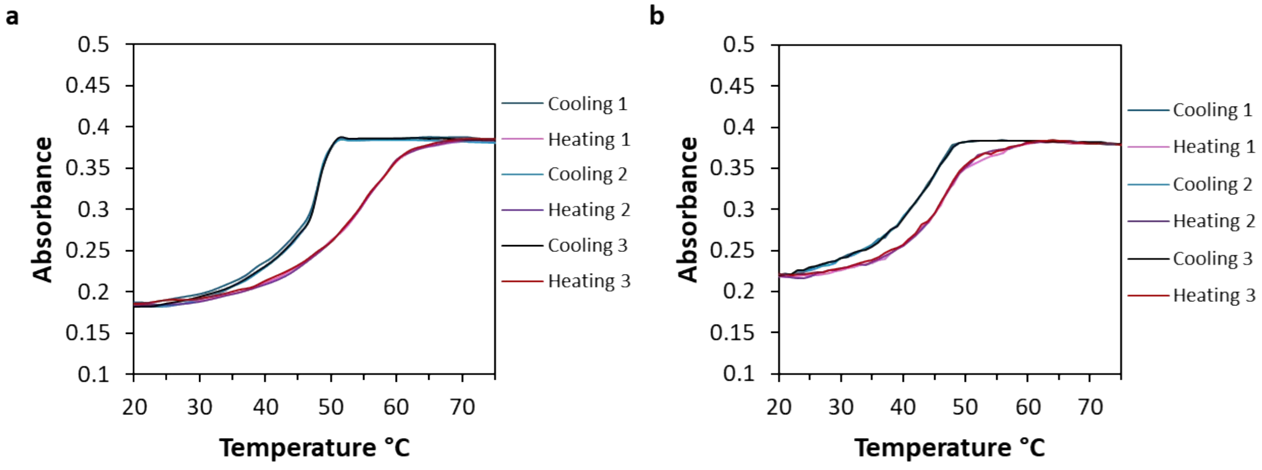

Figure 33 Reversibility of the self-assembly process is visualized by three consecutive UV-vis cooling and heating cycles for **8a** (a) and **8b** (b). Absorbance is monitored at 330 nm during cooling and heating from 75 °C to 20 °C, gradient: 0.5 °C/min.

6. Atomic force microscopy

6.1. Zoom in

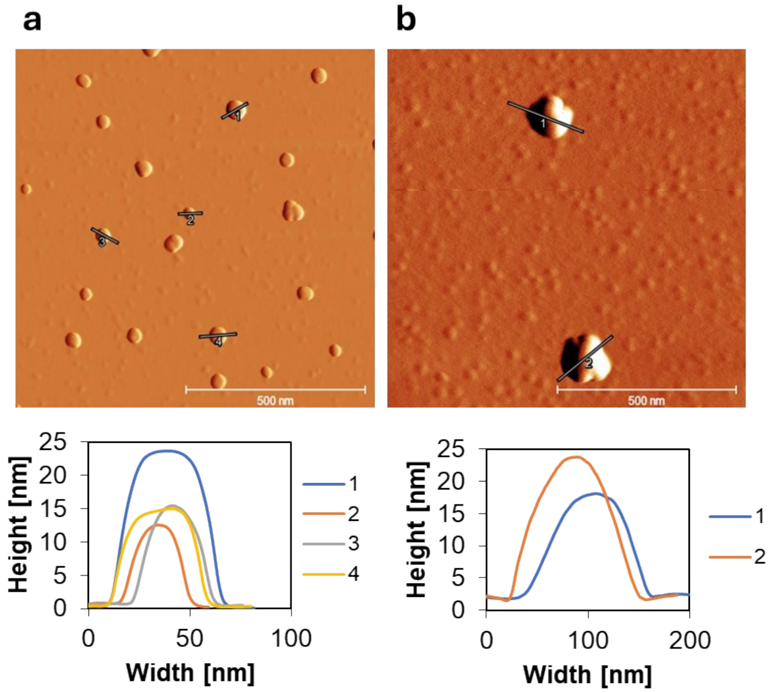

Figure 34 AFM images of **8a** (left) and **8b** (right).

6.2. Additional images

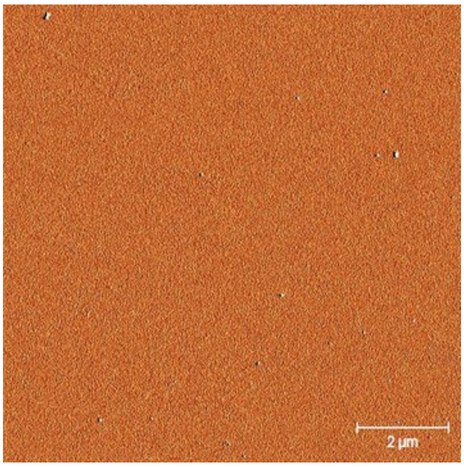

Figure 35 AFM image of a blank, Conditions: Aqueous solution with 10 mM sodium phosphate buffer pH 7.2, 100 mM NaCl, 10 vol% ethanol on APTES modified mica

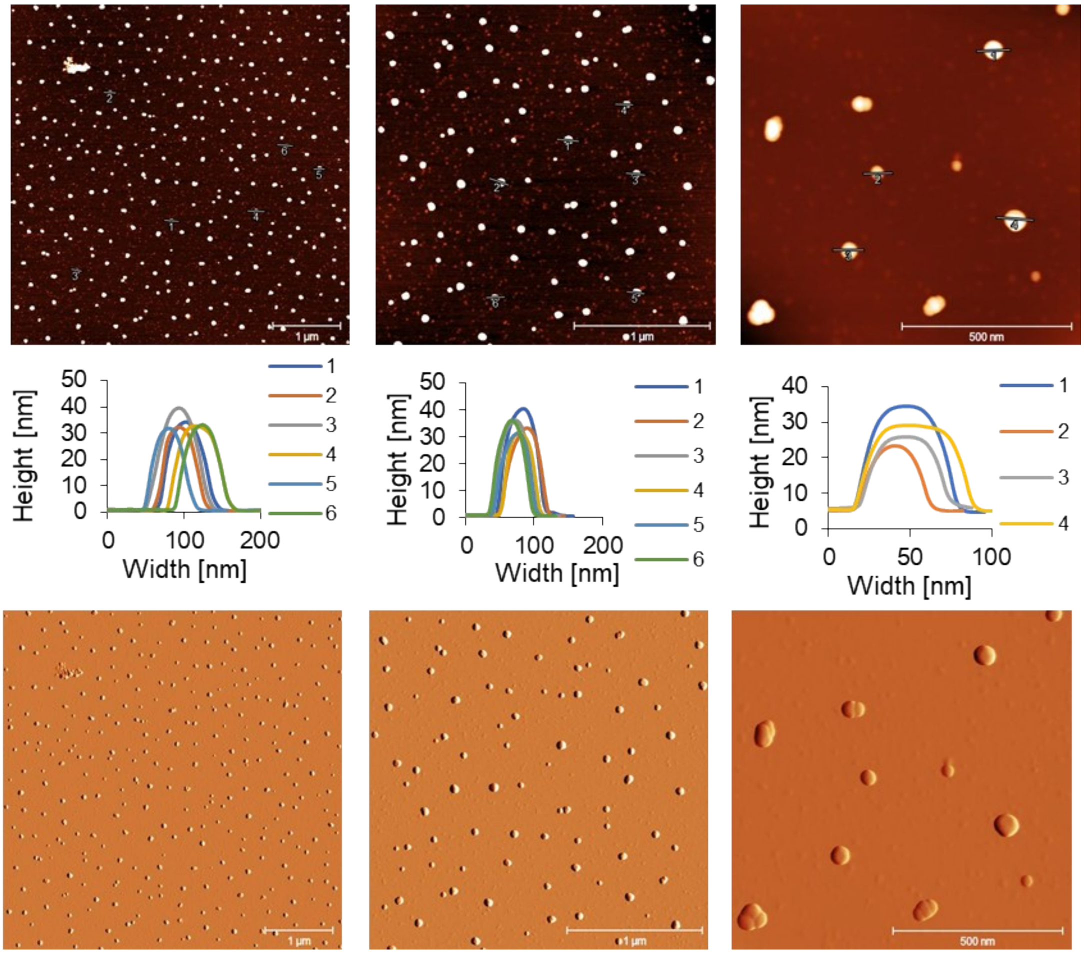

Figure 36 AFM images of **8a** with cross-sections. Above: Height-scan; Below: Amplitude scan.

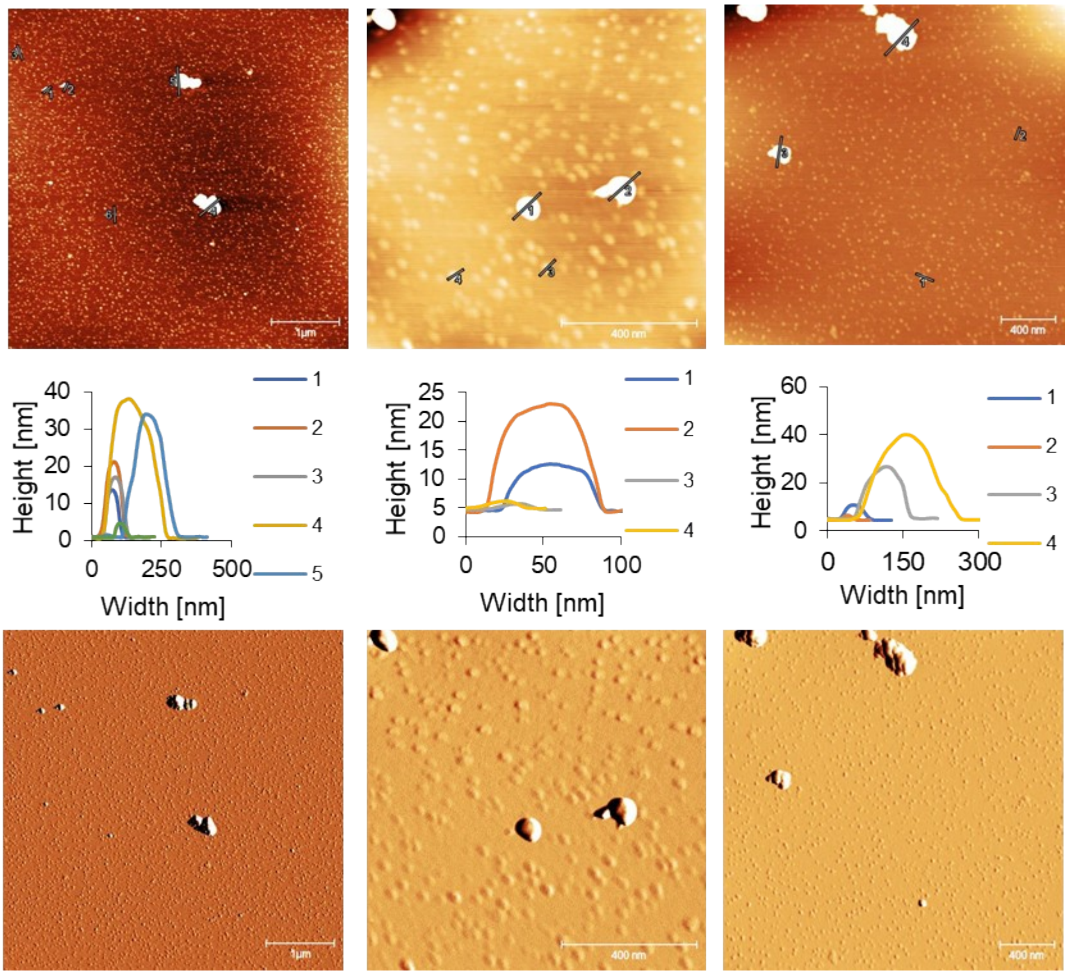

Figure 37 AFM images of **8b** with cross-sections. Above: Height-scan; Below: Amplitude scan.

7. Dynamic light scattering

In addition to AFM, DLS experiments of the supramolecular assemblies in solution were performed. Average diameters of 60 ± 12 nm for **8a** and 158 ± 29 nm for **8b** were measured. These findings are in good agreement with the AFM measurements of the assembled nanostructures (Table 1).

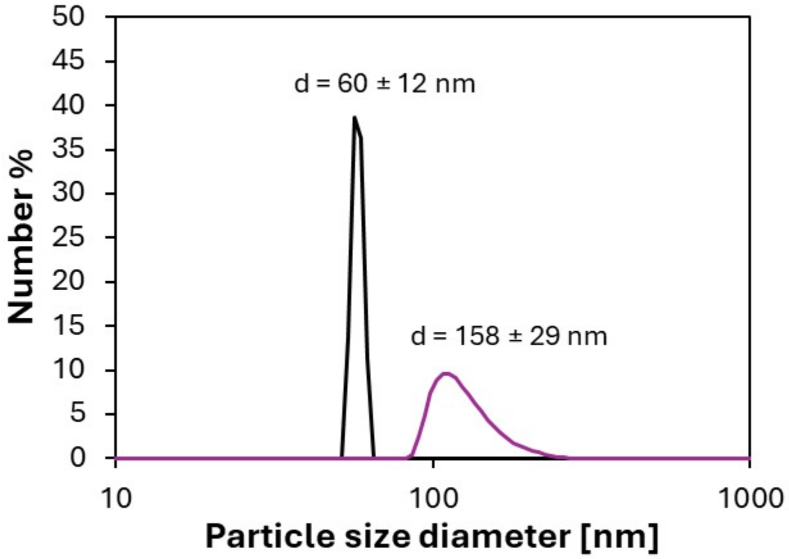

Figure 38 DLS measurements of **8a** (black) and **8b** (pink) showing the particle size distribution (hydrodynamic diameter) of the nanostructures at 20 °C. Conditions: Aqueous solution with 3 µM trimer, 10 mM sodium phosphate buffer pH 7.2, 100 mM NaCl, 10 vol% ethanol.

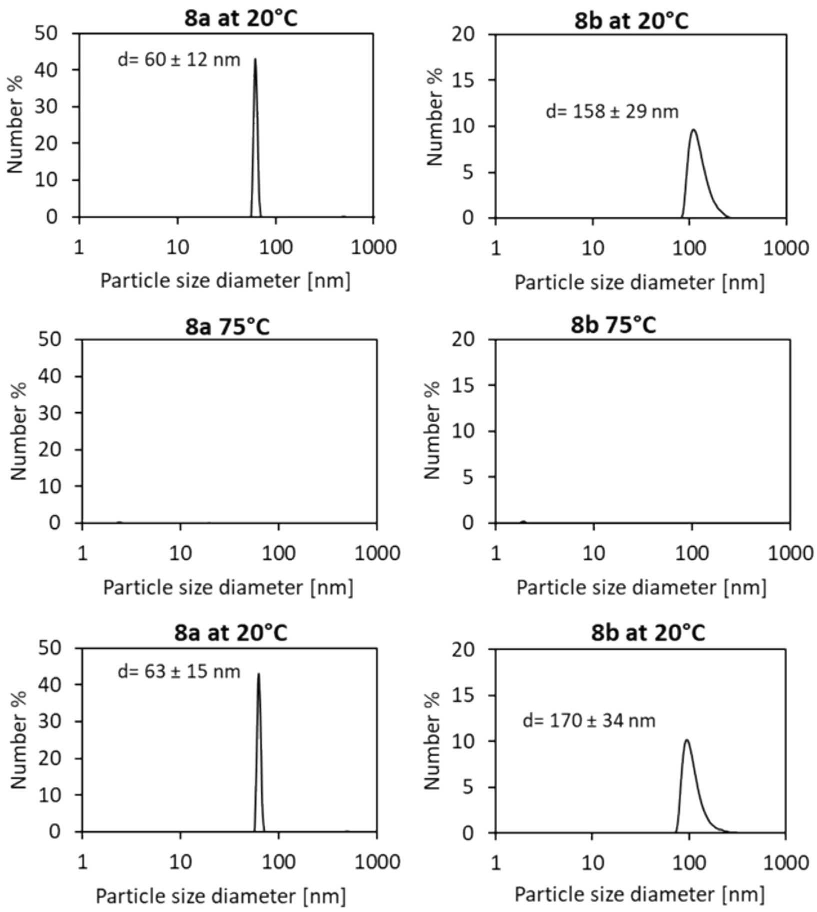

Figure 39 DLS measurements of 8a (left) and 8b (right). Self-assembled solutions of **8a** and **8b** were measured at 20°C, heated to 75°C (to ensure full disassembly), measured, and then cooled (0.5 °C/min) back to 20 °C and measured again. Conditions: Aqueous solution with 3 µM trimer, 10 mM sodium phosphate buffer pH 7.2, 100 mM NaCl, 10 vol% ethanol

	Size AFM [diameter]	Size DLS [diameter]	T_n UV-vis	T_n FL	Quantum Yield [%]
**8a** nanostructure	50 ± 17 nm	60 ± 12 nm	49°C	49°C	0.55%
**8b** nanostructure	166 ± 31 nm	158 ± 29 nm	47°C	44°C	0.12%

Table 1. Comparison of the obtained results and sizes. All measurements were performed at 20°C and in the same conditions.

8. References

1 T. Takeda and T. Akutagawa, *Chem. – Eur. J.*, 2016, **22**, 7763–7770.
2 W. Chen, S. Wang, G. Yang, S. Chen, K. Ye, Z. Hu, Z. Zhang and Y. Wang, *J. Phys. Chem. C*, 2016, **120**, 587–597.
3 S. Rothenbühler, I. Iacovache, S. M. Langenegger, B. Zuber and R. Häner, *Nanoscale*, 2020, **12**, 21118–21123.
4 Y. L. Lyubchenko, B. L. Jacobs and S. M. Lindsay, *Nucleic Acids Res.*, 1992, **20**, 3983–3986.
